# Supplementary material for: 2,6-diaminopurine promotes repair of DNA lesions under prebiotic conditions
Source: Nat Commun. 2021 May 21;12:3018. doi: 10.1038/s41467-021-23300-y (PMC8139960; doi:10.1038/s41467-021-23300-y)
Supplement: Supplementary file 1 — Supplementary Information [file 41467_2021_23300_MOESM1_ESM.pdf]

Supplementary Information:

2,6-diaminopurine promotes repair of DNA lesions under prebiotic conditions

Rafał Szabla,[1,2*] Magdalena Zdrowowicz,[3*] Paulina Spisz,[3] Nicholas J. Green,[4] Petr Stadlbauer,[5] Holger Kruse,[5] Jiří Šponer,[5] Janusz Rak[3]

[1]EaStCHEM School of Chemistry, University of Edinburgh, Joseph Black Building, David Brewster Road, Edinburgh, EH9 3FJ, UK.
[2]Institute of Physics, Polish Academy of Sciences, Al. Lotników 32/46, 02-668 Warsaw, Poland
[3]Faculty of Chemistry, University of Gdańsk,Wita Stwosza 63, 80-308 Gdańsk, Poland
[4]MRC Laboratory of Molecular Biology, Francis Crick Avenue, Cambridge Biomedical Campus, Cambridge, CB2 0QH, UK.
[5]Institute of Biophysics of the Czech Academy of Sciences, Kralovopolska 135, 61265 Brno, Czech Republic

*Correspondence to; E-mail: rafal.szabla@ed.ac.uk, magdalena.zdrowowicz@ug.edu.pl

This PDF file includes:

Materials and Methods
Supplementary Discussion
Supplementary Figures
Supplementary Tables
References

Contents

S1 Schematic representation of the DNA repair mechanism, as performed by photolyases 3
S2 Supplementary Results and Discussion 4
S2.1 Experimental Results 4
Solid-state glycosylation of 2,6-diamonopurine (Dap) with 2'-deoxy-2-thiouridine (2tU) to form β-2,6-diaminopurine 2′-deoxyriboside (β-D) – spectra 4
Generation of damaged trinucleotides 6
Identification of studied trinucleotides – LC-MS and LC-MS/MS analysis 7
Photostationary equilibria 18
Quantum Yields 19
Irradiation conditions compared to the UV environment of early Earth 20
UV-irradiation experiments of aqueous adenine and 2,6-diaminopurine deoxyribosides (A and D) 21
S2.2 Computational Results 23
Ionization energies 23
MD simulations and conformational analysis of damaged DNA oligomers 24
Ground-state geometries of the selected DNA trimers 27
Vertical excitation energies of the studied DNA trimers 28
Electron transfer rates calculated using a quasi-Marcus approach 29
Characteristics of the CT states in DNA trimers 32
Opening of the T=T dimer after photoinduced electron transfer 33
Excited-state QM/MM calculations for the ADT=TA pentamer 35
Supplementary References 37

S1 Schematic representation of the DNA repair mechanism, as performed by photolyases.

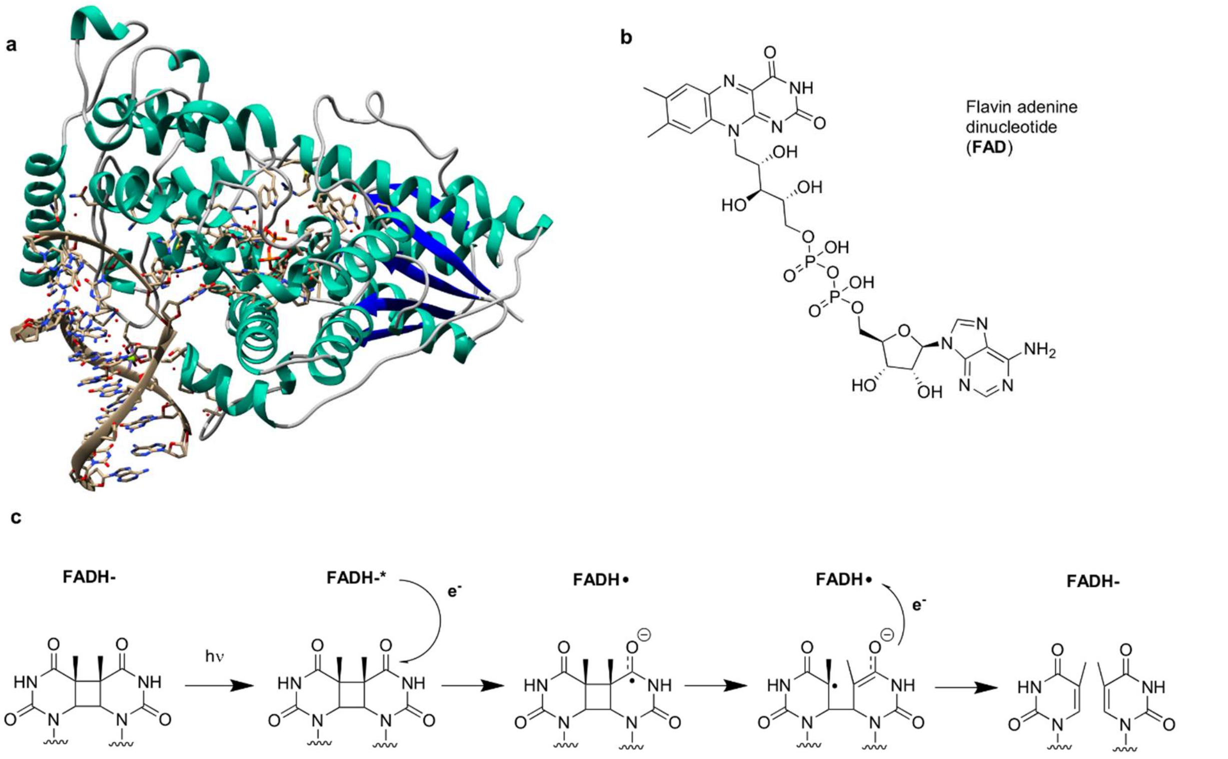

Supplementary Figure 1. Molecular mechanism of photoinduced repair of CPDs by photolyases. **a,** structure of the crystallized complex between the *Anacystis nidulans* DNA photolyase and a 14-nucleotide DNA oligomer after performing repair of the T=T dimer (PDB: 1TEZ from Ref. 7 in the main article). **b,** structure of the flavin adenine dinucleotide (FAD), the photoredox active enzyme cofactor responsible for transferring electrons and conducting CPD repair. **c,** molecular mechanism of CPD repair induced by electron transfer from the reduced and negatively charged form of FAD, *i.e.* FADH-.

S2 Supplementary Results and Discussion

S2.1 Experimental Results

Solid-state glycosylation of 2,6-diamonopurine (Dap) with 2′-deoxy-2-thiouridine (2tU) to form β-2,6-diaminopurine 2′-deoxyriboside (β-D) – spectra.

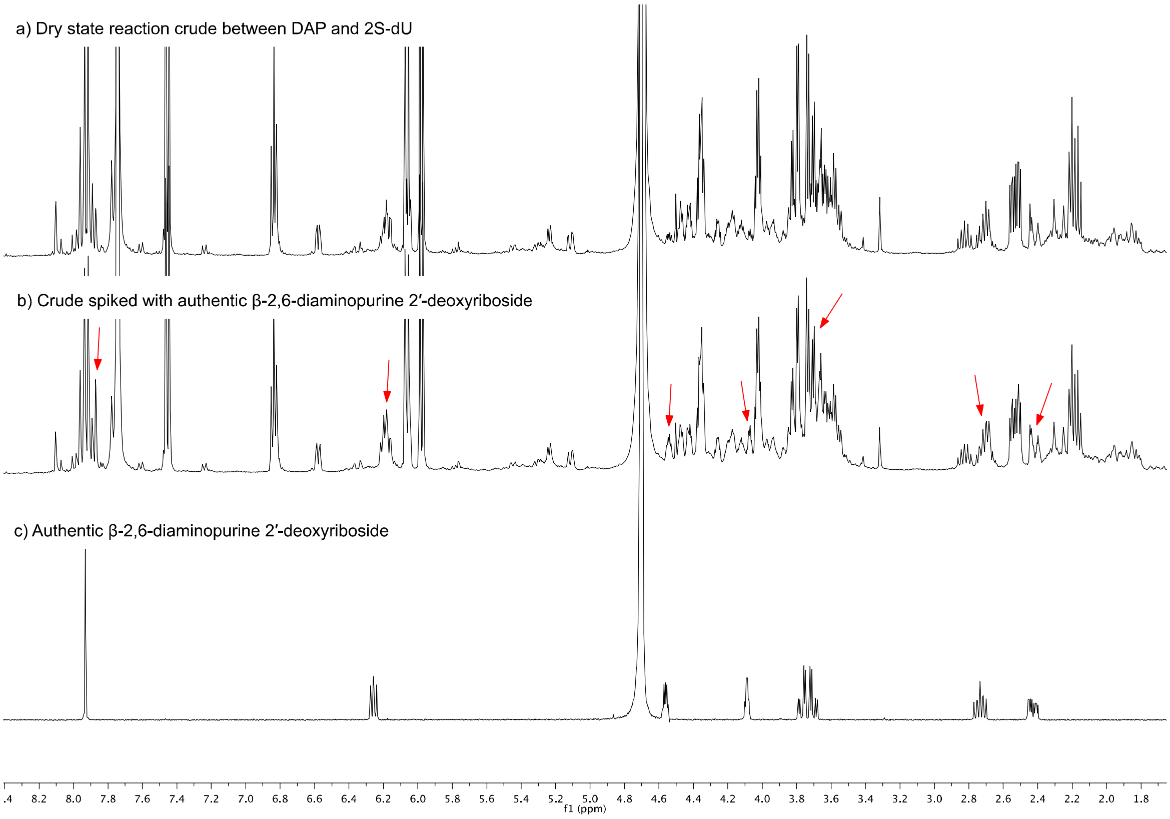

Supplementary Figure 2. Supplementary spectra for the synthesis of β-2,6-diaminopurine 2′-deoxyriboside. 1H NMR of a) crude glycosylation mixture; b) crude spiked with **β-D**; and c) pure **β-D**.

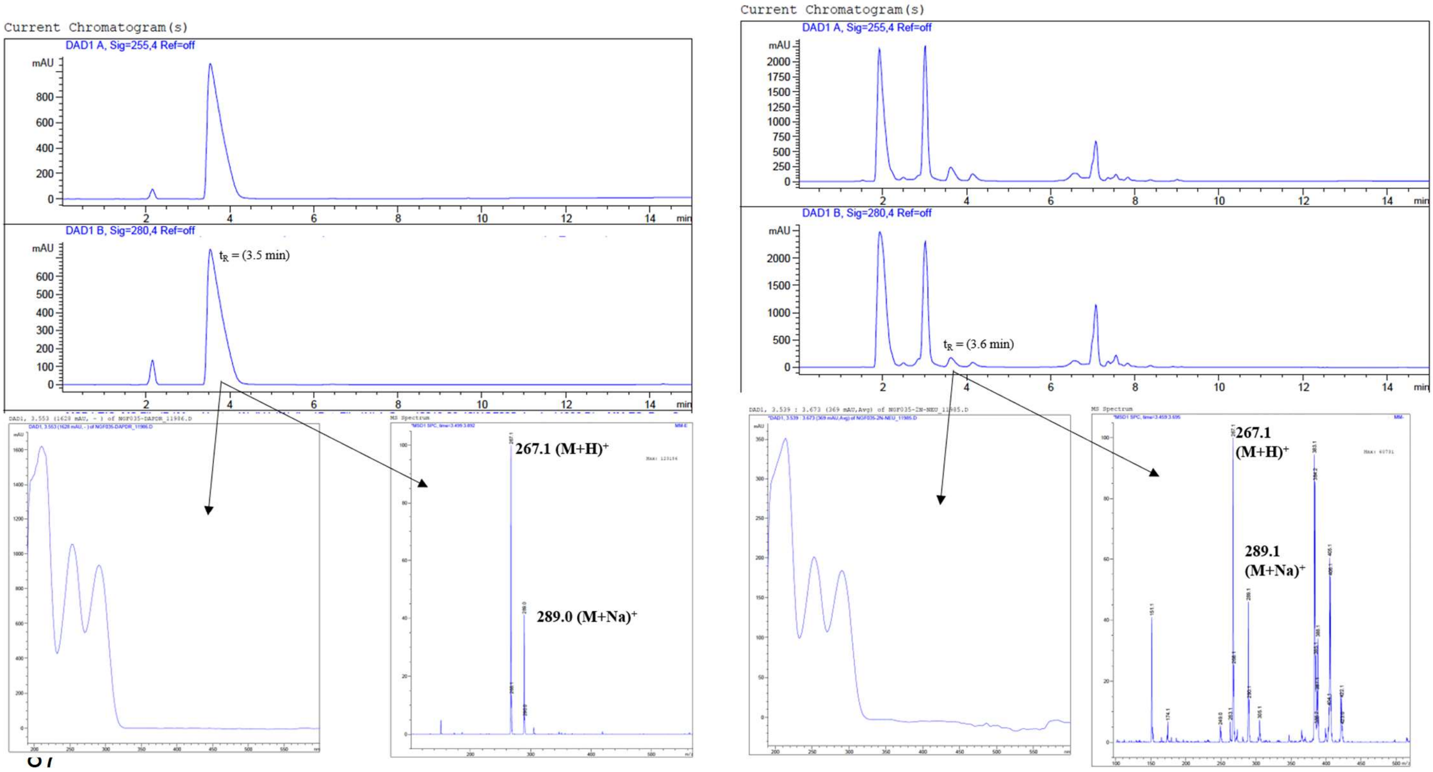

Supplementary Figure 3. LCMS and UV-Vis spectrum of pure **β-D**, left; and crude glycosylation mixture, right. The product of glycosylation shows the same retention time, mass spectrum, and UV-Vis spectrum as the authentic material.

Generation of damaged trinucleotides

All trinucleotides containing thymidine dimer were obtained photochemically by irradiation with UVB light (see *Generation of AT=T, T=TA, DT=T, T=TD* in methods section). The photoproduct was purified and accumulated by HPLC. The efficiency of damaged trinucleotide generation was significantly higher in the case of sequences ATT and TTA (see Supplementary Figure 4).

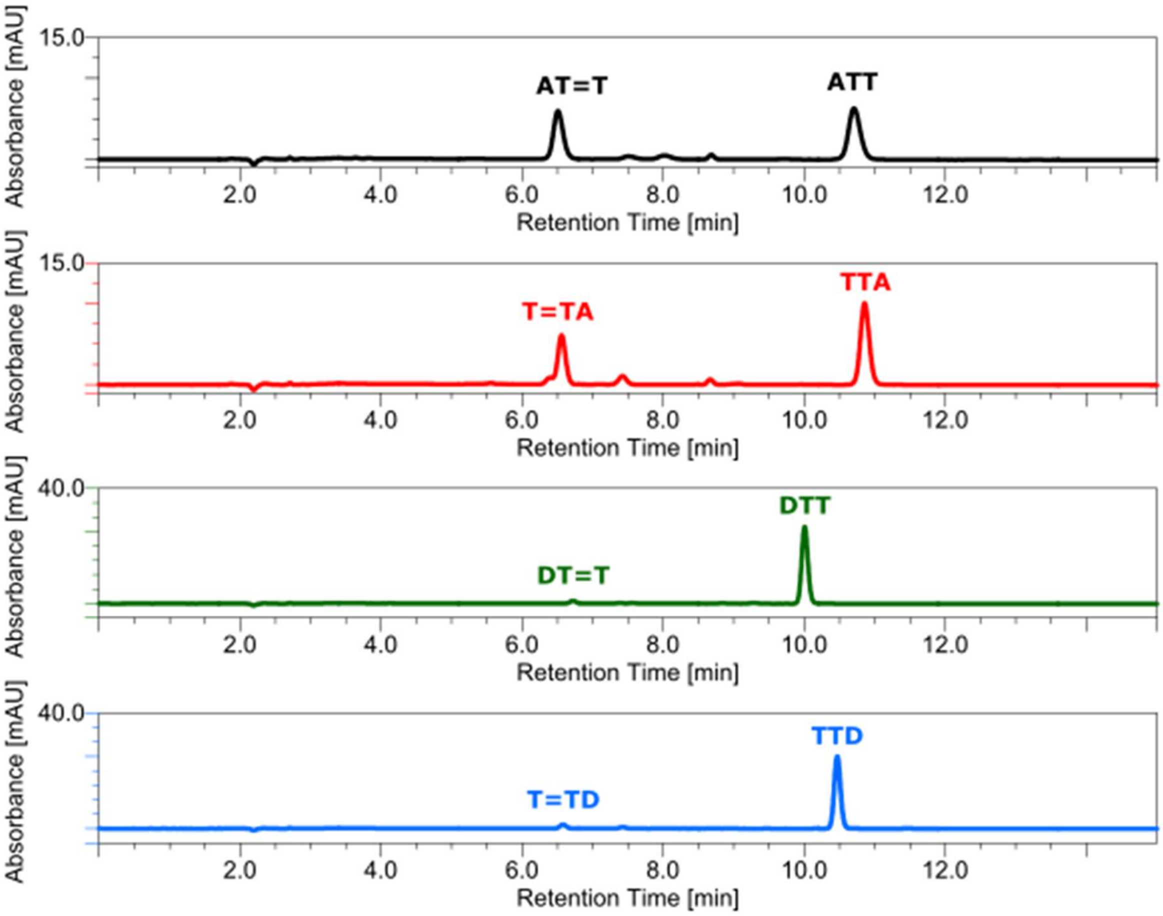

Supplementary Figure 4. HPLC traces of studied trinucleotides: ATT (black), TTA (red), DTT (green) and TTD (blue) after UV irradiation in 10 mM phosphate buffer containing 100 mM sodium chloride

Identification of studied trinucleotides – LC-MS and LC-MS/MS analysis

All studied trinucleotides (damaged and repaired) were identified by mass spectrometry (LC-MS and LC-MS/MS experiments). Supplementary Table 1 shows the theoretical and experimental *m/z* values for the studied sequences and MS spectra are presented in Supplementary Figures 5-8. All trinucleotides were detected as doubly charged ($[M - 2H]^{2-}$) species and singly charged ones ($[M - H]^{-}$). For example, in the case of ATT, the *m/z* 858.3921 corresponds to $[M - H]^{-}$ ion and *m/z* 428.6920 refers to $[M - 2H]^{2-}$ ion. The structural characteristics have been provided by tandem mass spectrometry. Tandem mass spectrometric experiments were performed on all of the singly charged deprotonated trinucleotides produced via electrospray ionization. The product-ion spectra of undamaged and damaged trinucleotides with ion identities are presented in Supplementary Figures 9-17. The fragmentation pathways characteristic for oligonucleotide are observed and involve the loss of thymine/purine base, nucleoside or dinucleotide. The MS/MS spectra of damaged sequences differ from these undamaged ones. The main difference is that the thymine is not lost, remaining linked to the adjacent base. Generally, in the case of trinucleotides containing thymidine dimer, the lack of signals of all product-ions generated on fragmentation pathways related with the elimination of a single thymine, can be observed. In addition, the identification of trinucleotides was based on the UV spectra, which, together with the marked maxima, are presented in Supplementary Figure 17.

Supplementary Table 1. M/z values for studied trinucleotides determined by ESI mass spectrometry.

Sequence	$[M-1]^-$ calculated	$[M-1]^-$ found
ATT	858.1866	858.3921
AT=T	858.1866	858.3912
TTA	858.1866	858.3870
T=TA	858.1866	858.3865
DTT	873.1975	873.4187
DT=T	873.1975	873.4187
TTD	873.1975	873.4122
T=TD	873.1975	873.4115

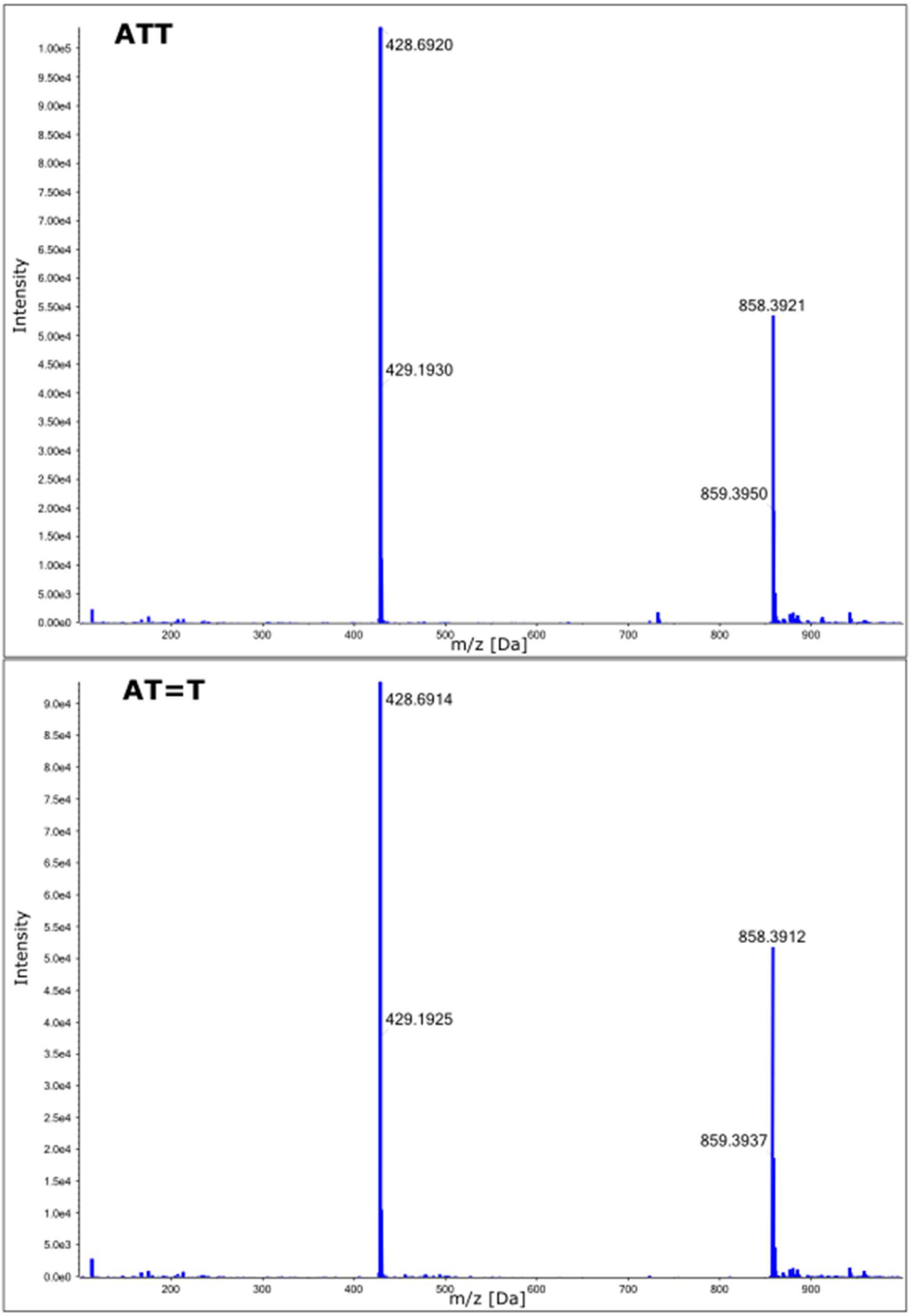

Supplementary Figure 5. MS spectra of ATT and AT=T.

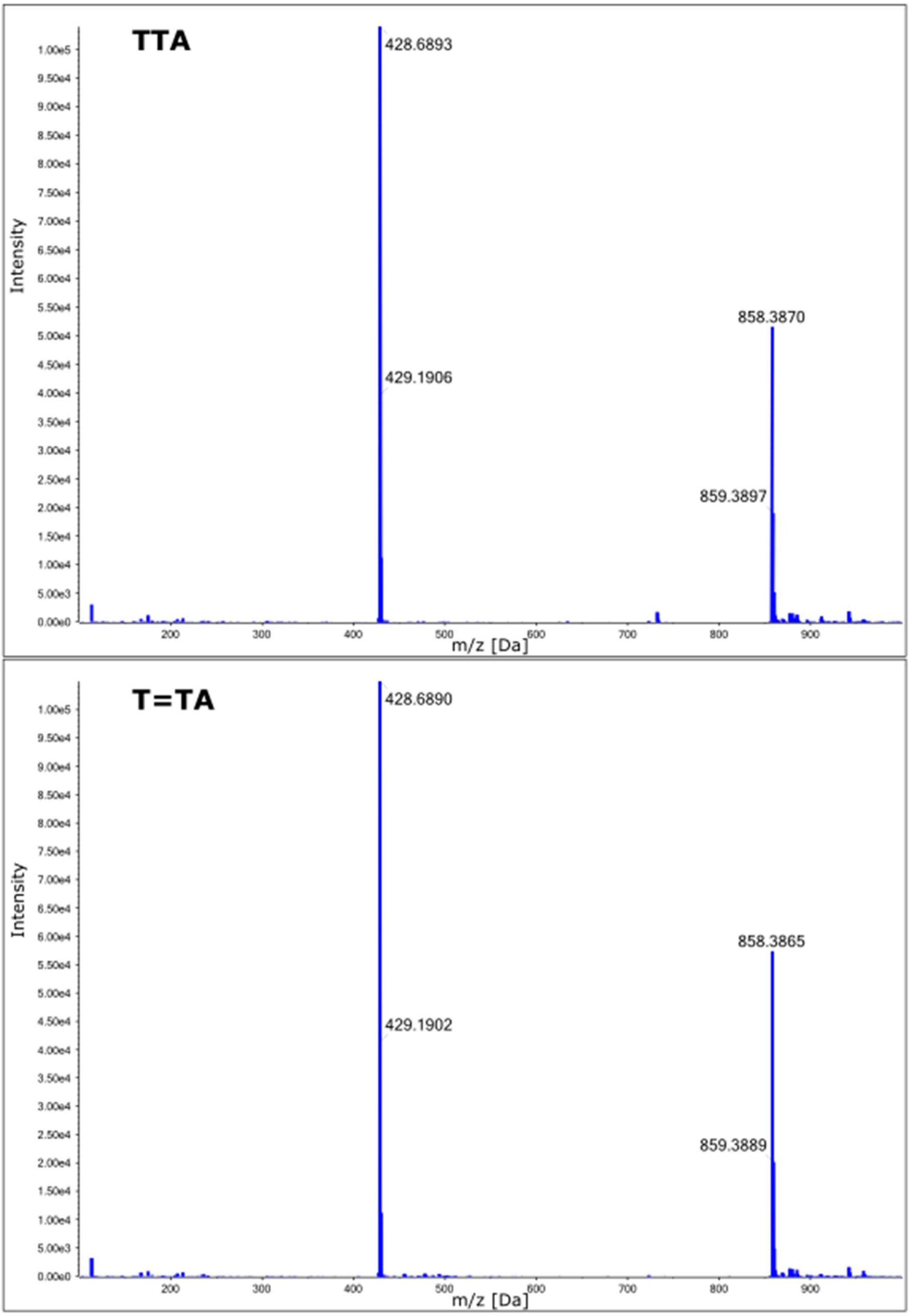

Supplementary Figure 6. MS spectra of TTA and T=TA.

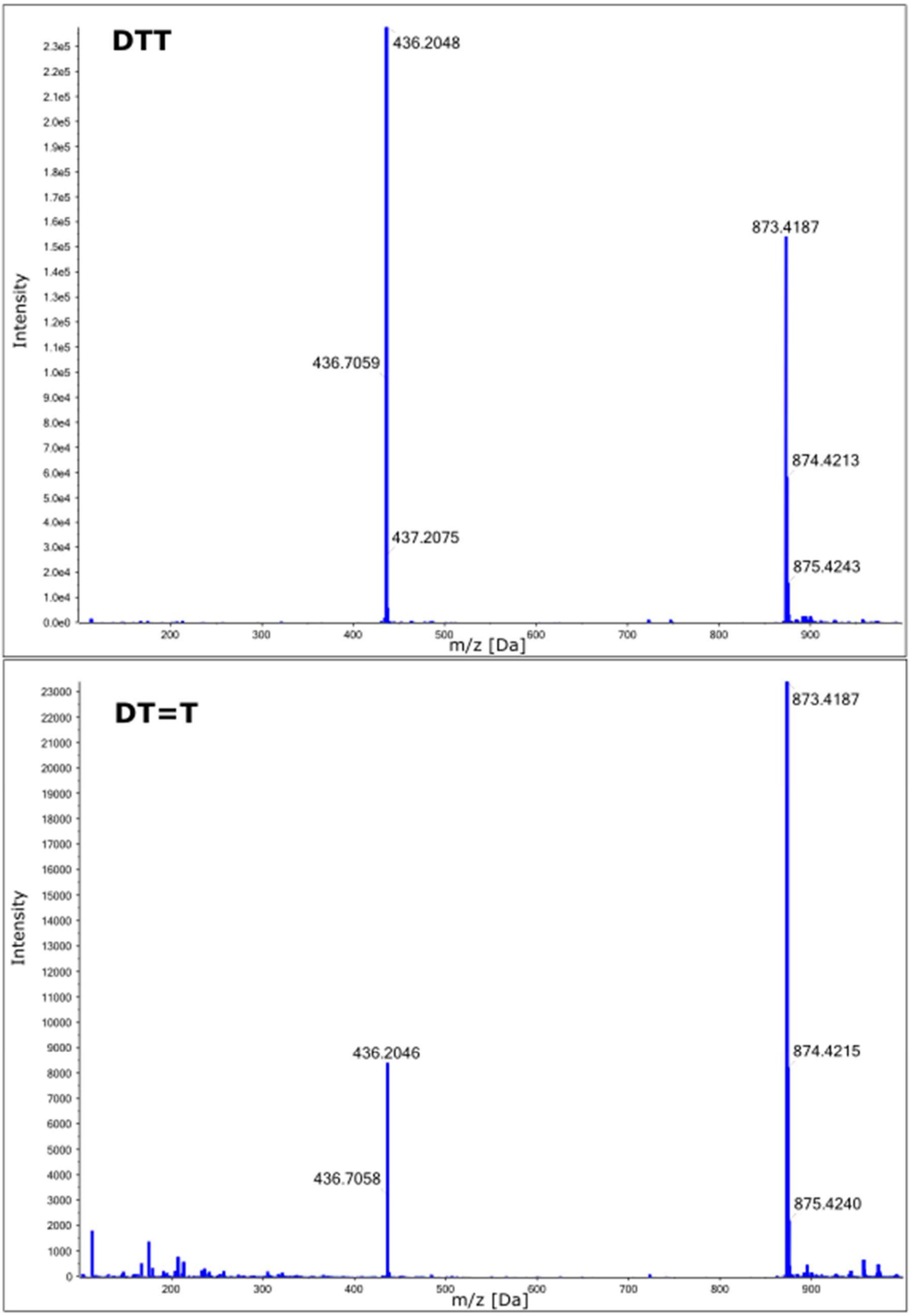

Supplementary Figure 7. MS spectra of DTT and DT=T.

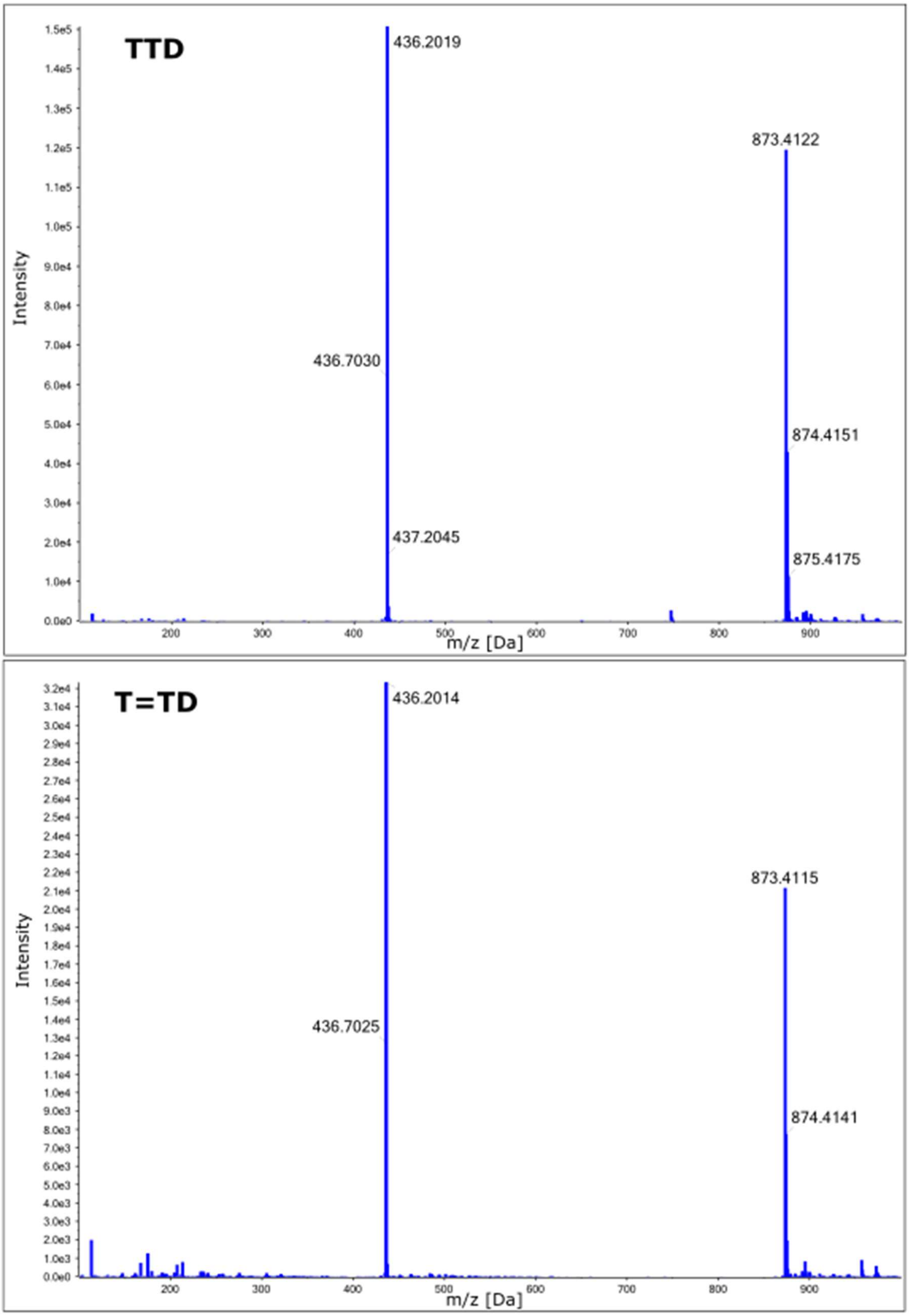

Supplementary Figure 8. MS spectra of TTD and T=TD.

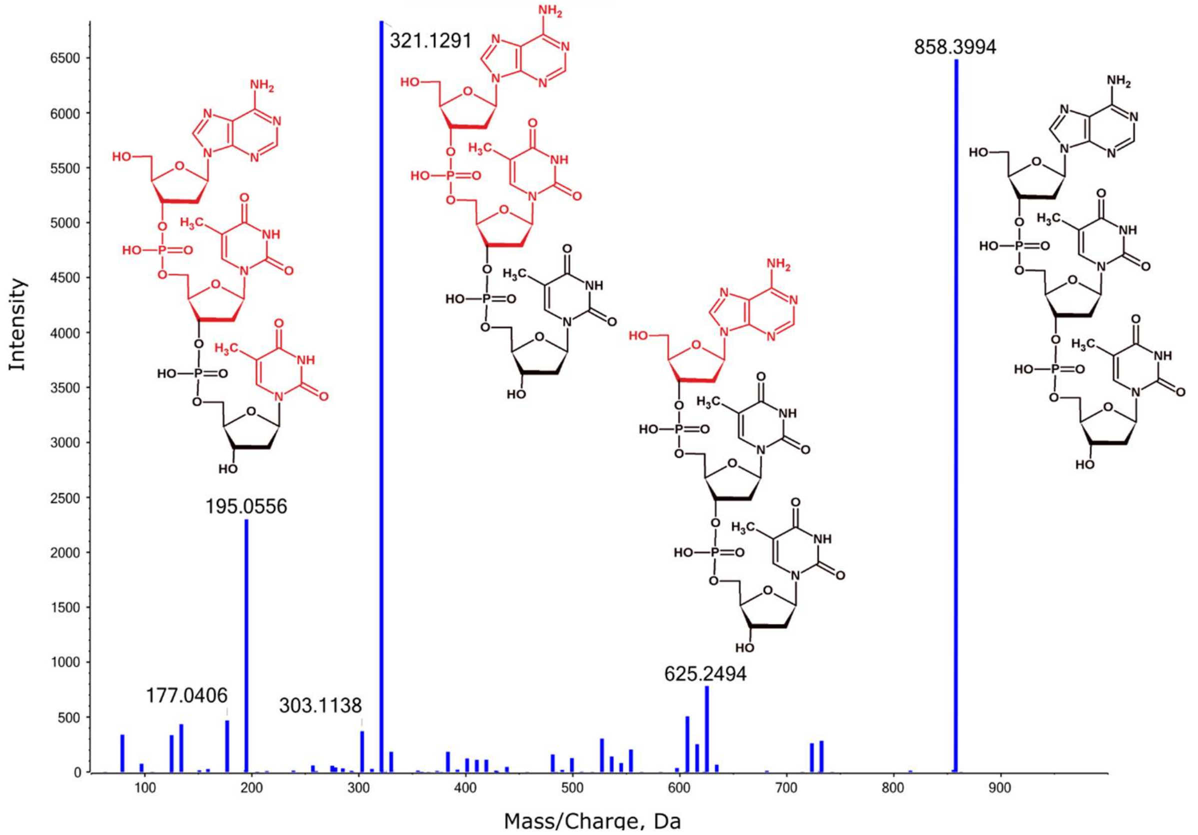

Supplementary Figure 9. MS/MS spectrum of ATT with ion identities.

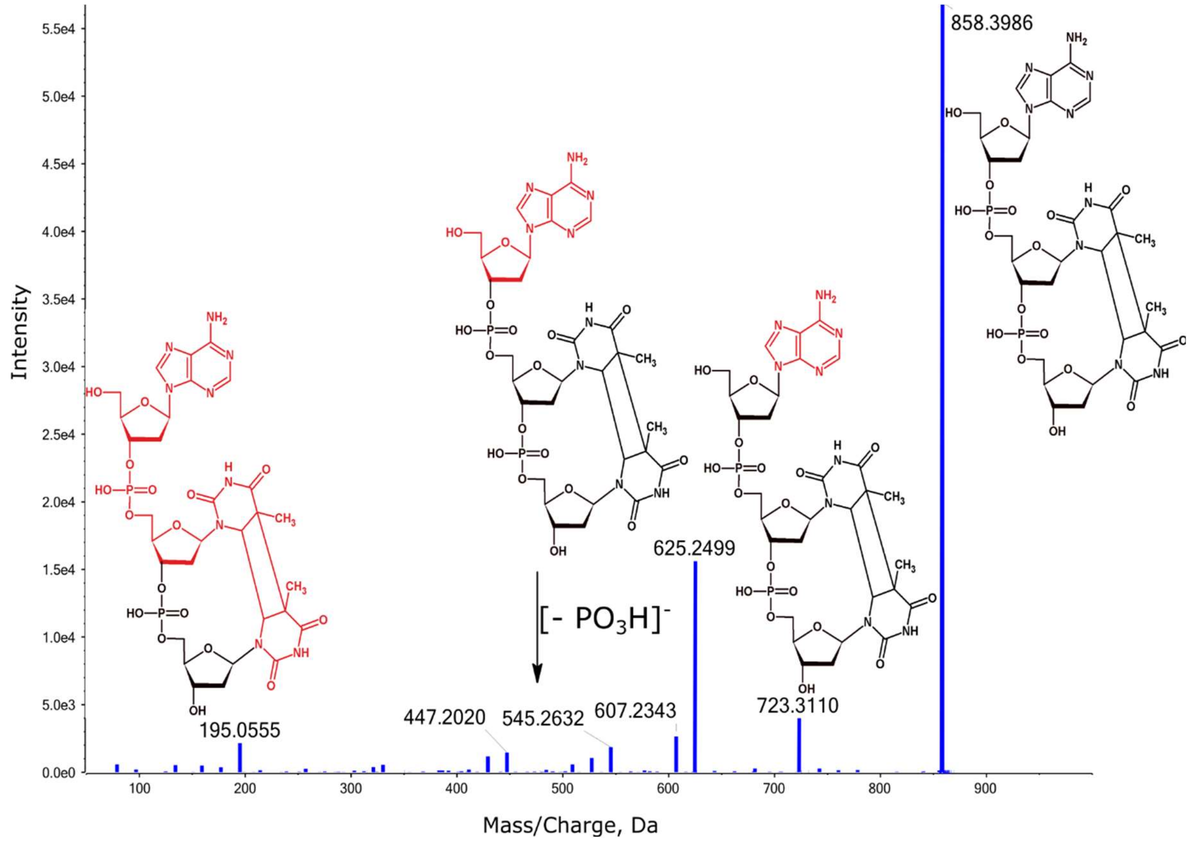

Supplementary Figure 10. MS/MS spectrum of AT=T with ion identities.

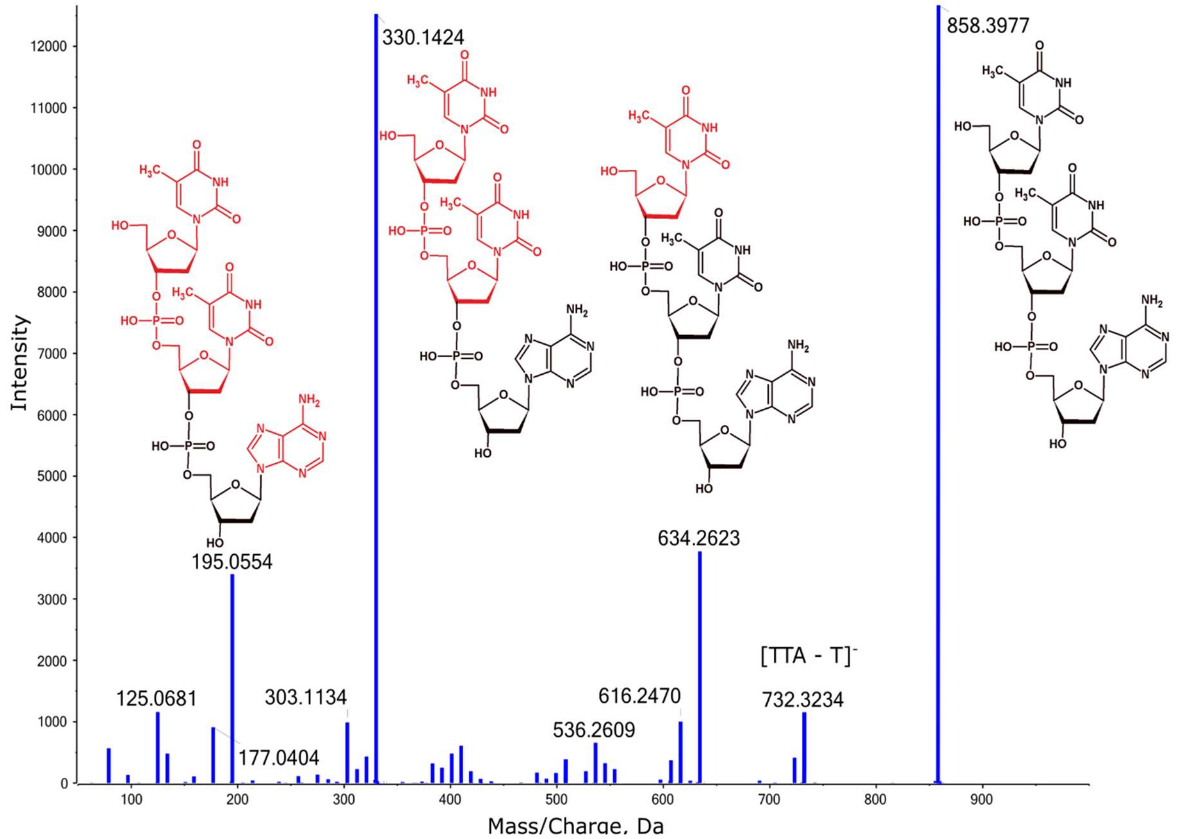

Supplementary Figure 11. MS/MS spectrum of TTA with ion identities.

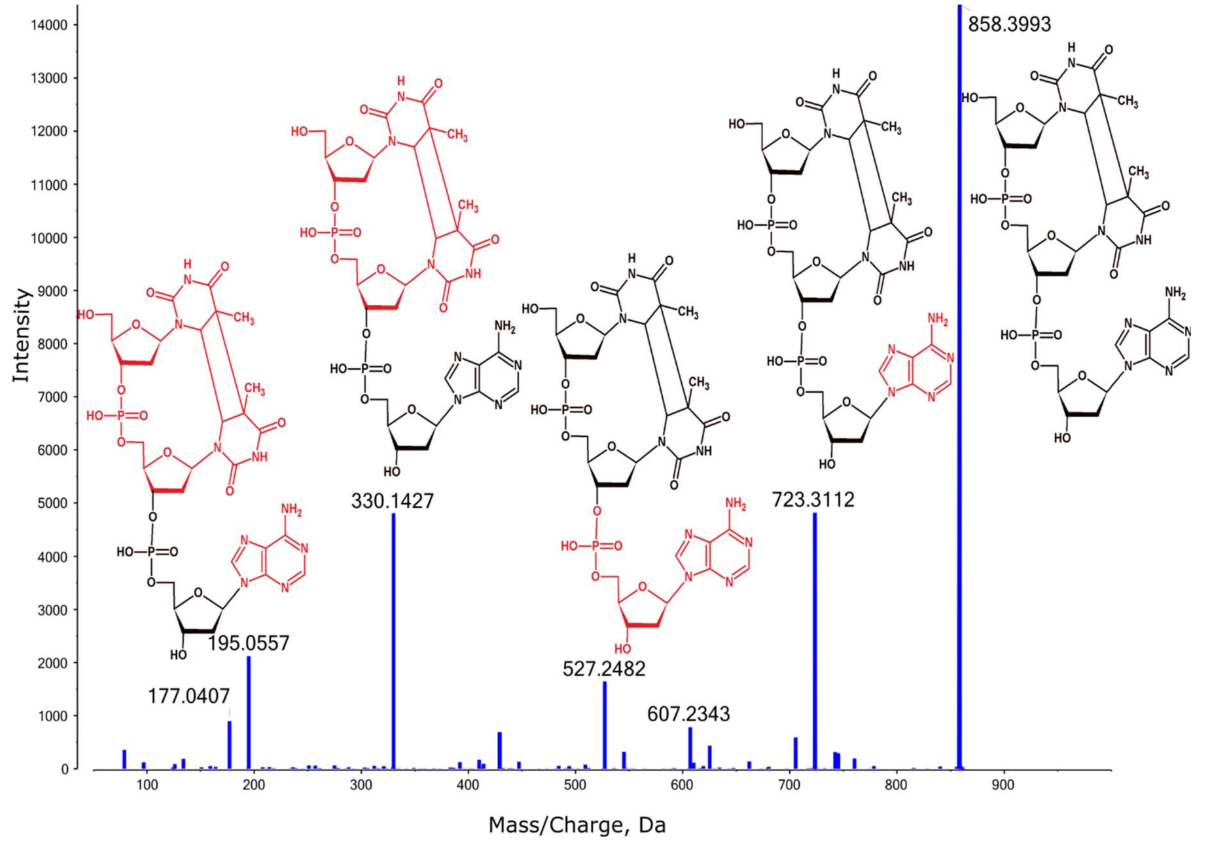

Supplementary Figure 12. MS/MS spectrum of T=TA with ion identities.

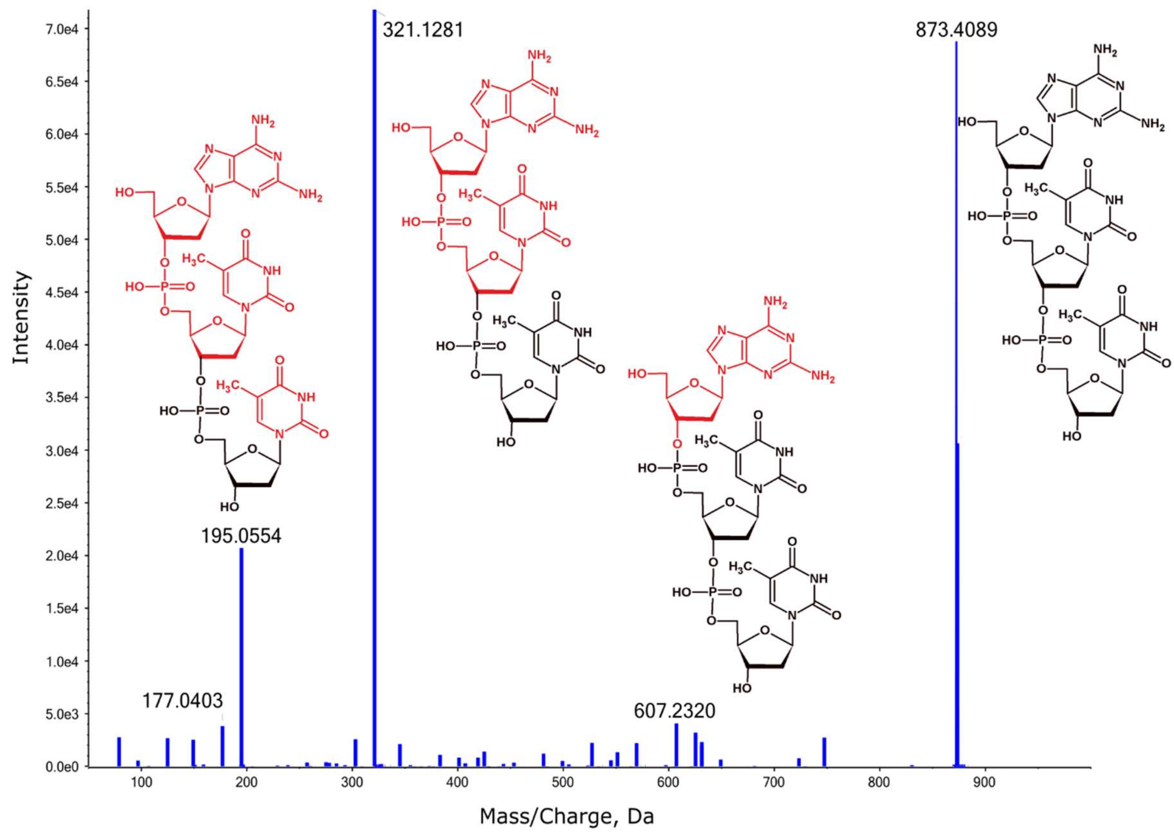

Supplementary Figure 13. MS/MS spectra of DTT with ion identities.

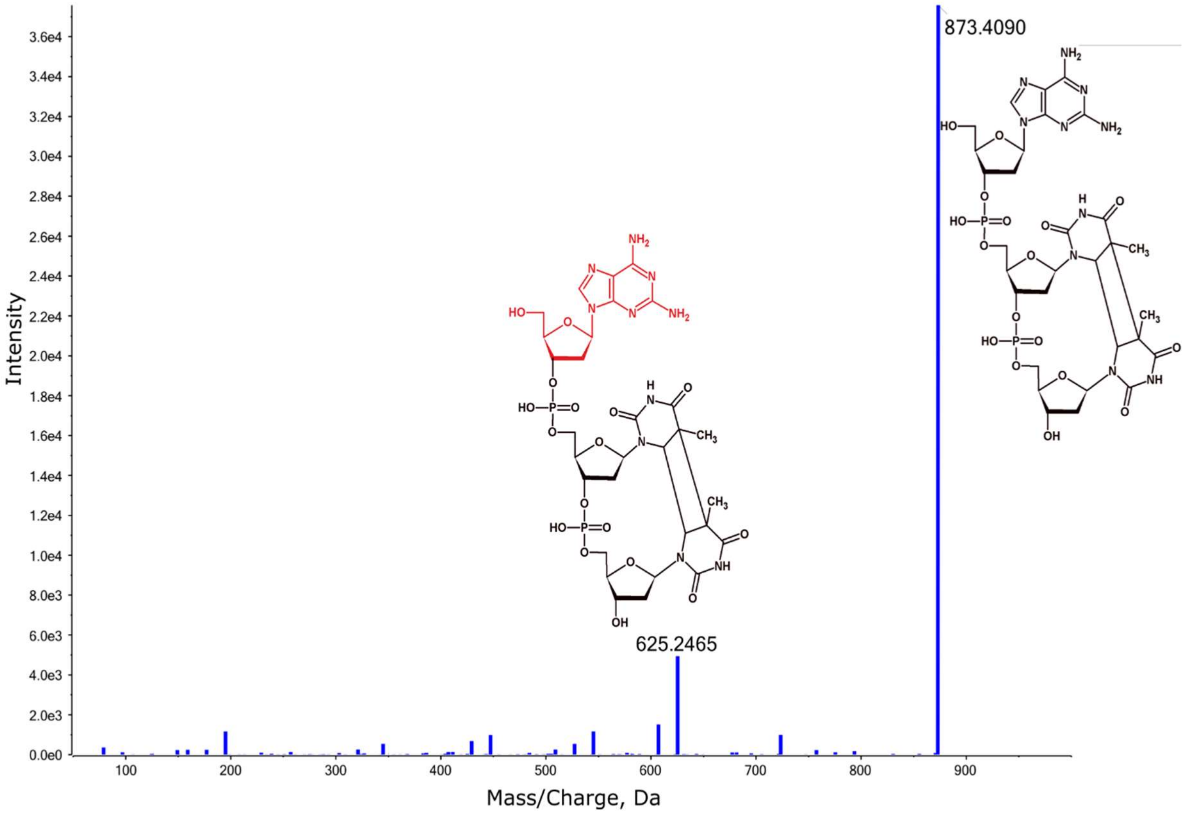

Supplementary Figure 14. MS/MS spectrum of DT=T with ion identities.

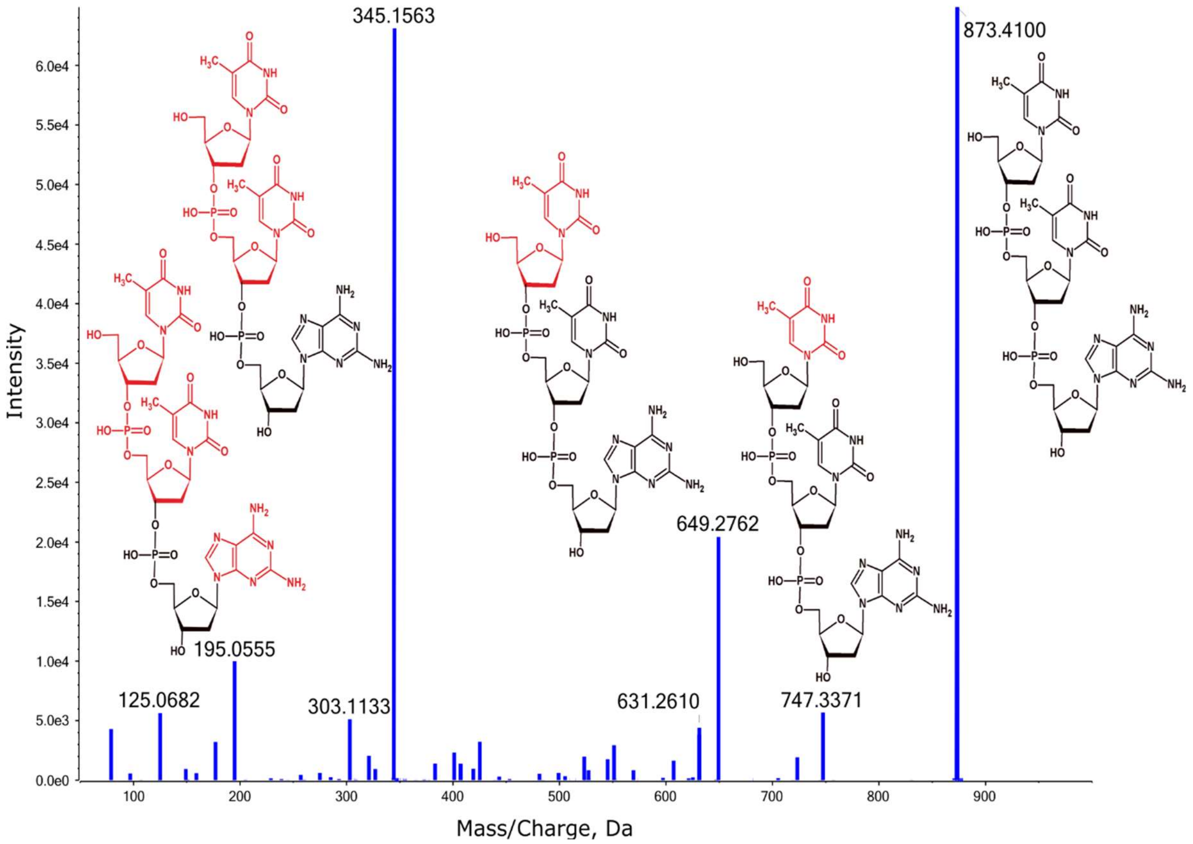

Supplementary Figure 15. MS/MS spectrum of TTD with ion identities.

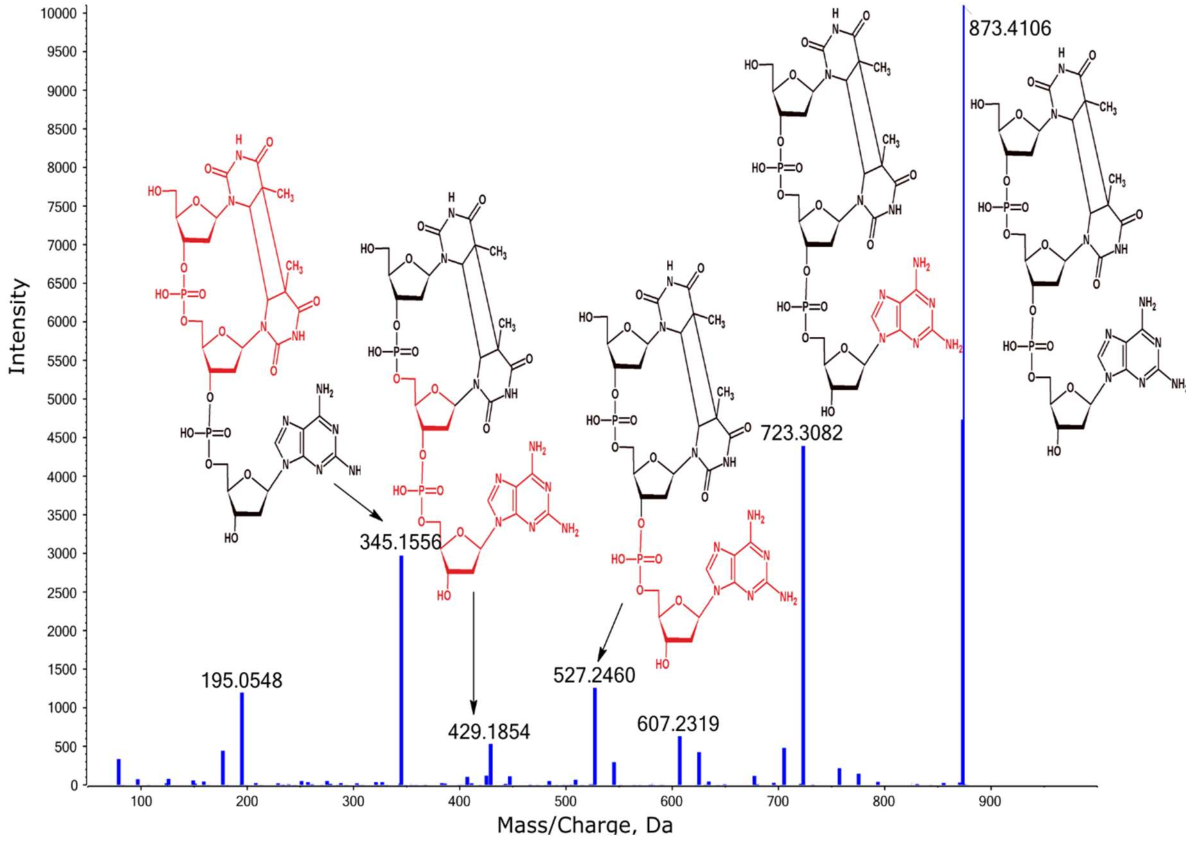

Supplementary Figure 16. MS/MS spectrum of T=TD with ion identities.

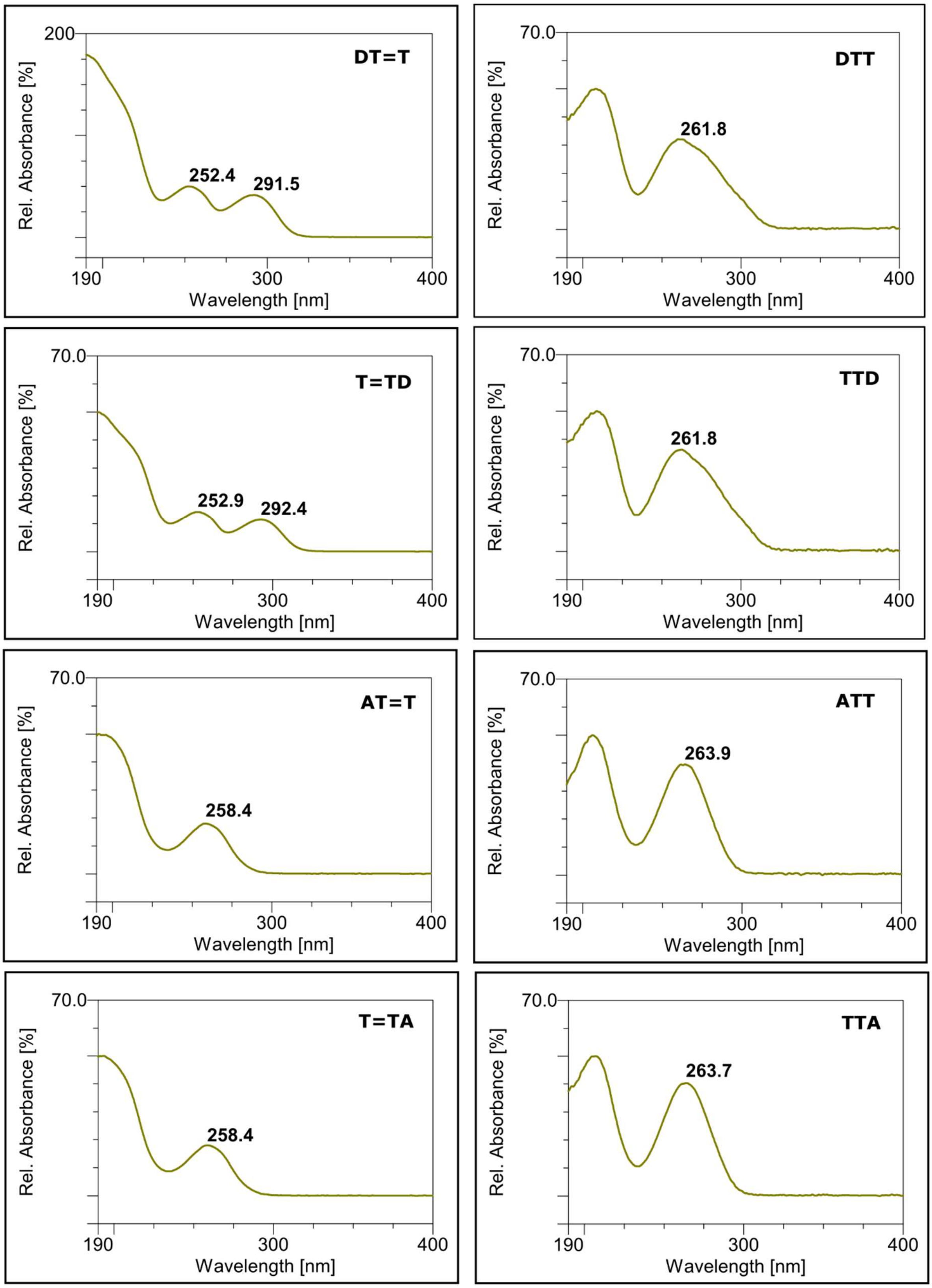

Supplementary Figure 17. UV spectra of studied trinucleotides.

Photostationary equilibria

Photostationary states were analyzed by HPLC analyses. A sample containing 20 µM trinucleotide in 10 mM sodium phosphate buffer (pH 7.0) was irradiated with UVB light (1.95 mW) in different time intervals (until the photostationary state was reached) and then chromatographed (Dionex Ultimate 3000 System; eluting condition: 0–25 B% in 15 min, B - 80% CAN, A - 0.1% HCOOH; C18 column Wakopak Handy ODS, 4.6 x 150 mm; 5 µm, 100 Å; monitoring wavelength 260 nm). % of conversion into the damaged trinucleotides was calculated by integration of the respective peak areas.

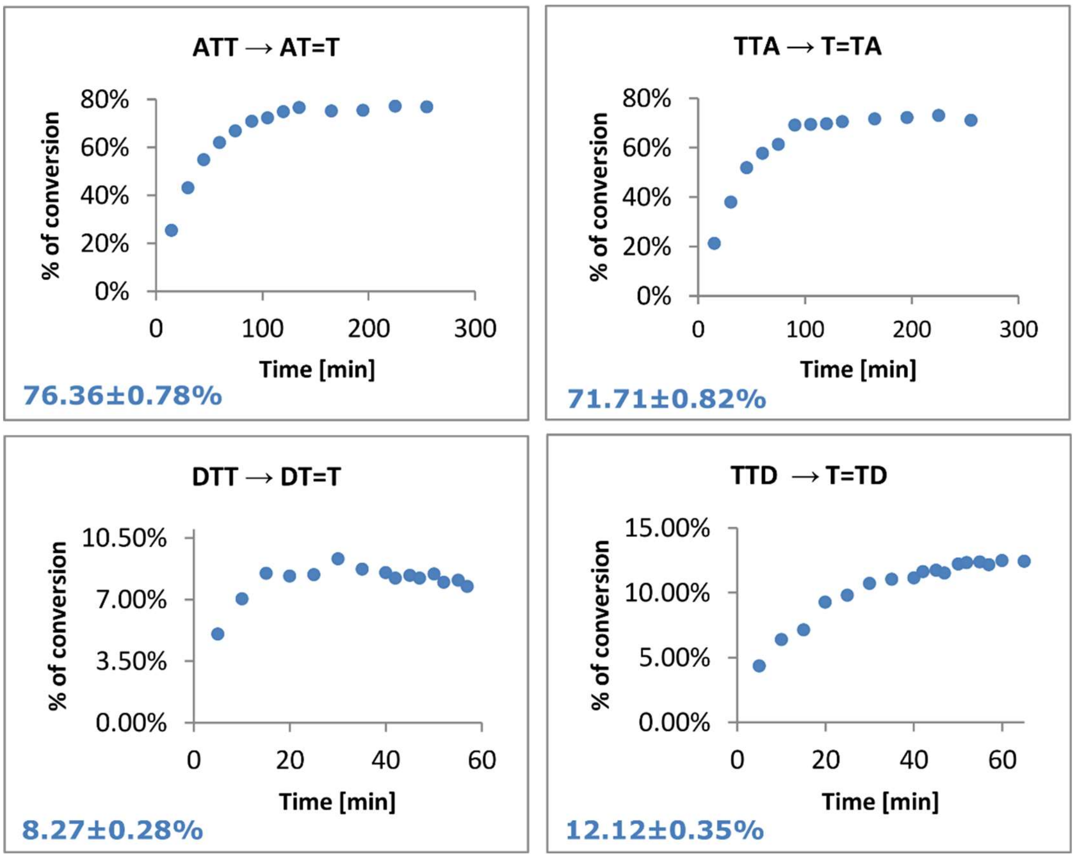

Supplementary Figure 18. Estimation of photostationary states of photodimerization in studied systems.

Quantum Yields

Quantum yields for dimer cleavage, Φ, were calculated as ratio of repaired molecules and photons absorbed by the system. The reported values were determined for the data after 45 min of irradiation (the smallest cumulative errors from measurements and HPLC analysis). Additionally, the conditional quantum yields taking into account the probability of exciting A or D, $\Phi_{A/D}$, were estimated. The resulting values for the dimer-containing trinucleotides are gathered in Supplementary Table 2. It is worth noting that the self-repair quantum yields determined for AT=T and T=TA are in excellent agreement with those determined by Pan et al.[1] under equivalent irradiation conditions.

Supplementary Table 2. Cleavage quantum yields, Φ, for thymine dimers in the listed sequences at 280 nm.

Sequence	Φ	$\Phi_{A/D}$[a]
AT=T	0.008625	0.009070
T=TA	0.006895	0.007250
DT=T	0.067002	0.067816
T=TD	0.063654	0.064427

[a] $\Phi_{A/D} = \Phi/P(A/D)$, where the probability of exciting A or D, P(A/D), is estimated from the molar absorption coefficients.[1]

Irradiation conditions compared to the UV environment of early Earth

Based on the meter readings (in W·cm^{-2}) for each set of UV-irradiation experiments we have estimated the absolute UV flux delivered at each irradiation wavelength considered in this work:

Irradiation chamber (irradiation of deoxyribonucleosides):
170 μW·cm^{-2} – 170·10^{-6} J s^{-1} cm^{-2} – **1700 erg s^{-1} cm^{-2}** (UVC, 254 nm)

Tunable UV lamp (irradiations of DNA trimers):
80 μW·cm^{-2} – 80·10^{-6} J s^{-1} cm^{-2} – **800 erg s^{-1} cm^{-2}** (280 nm)

Considering that the integrated surface flux between 200 and 300 nm delivered to the surface of early Earth amounts to 2700 erg s^{-1} cm^{-2},[2] we anticipate that our irradiation conditions are relevant for the prebiotic environment.

UV-irradiation experiments of aqueous adenine and 2,6-diaminopurine deoxyribosides (A and D)

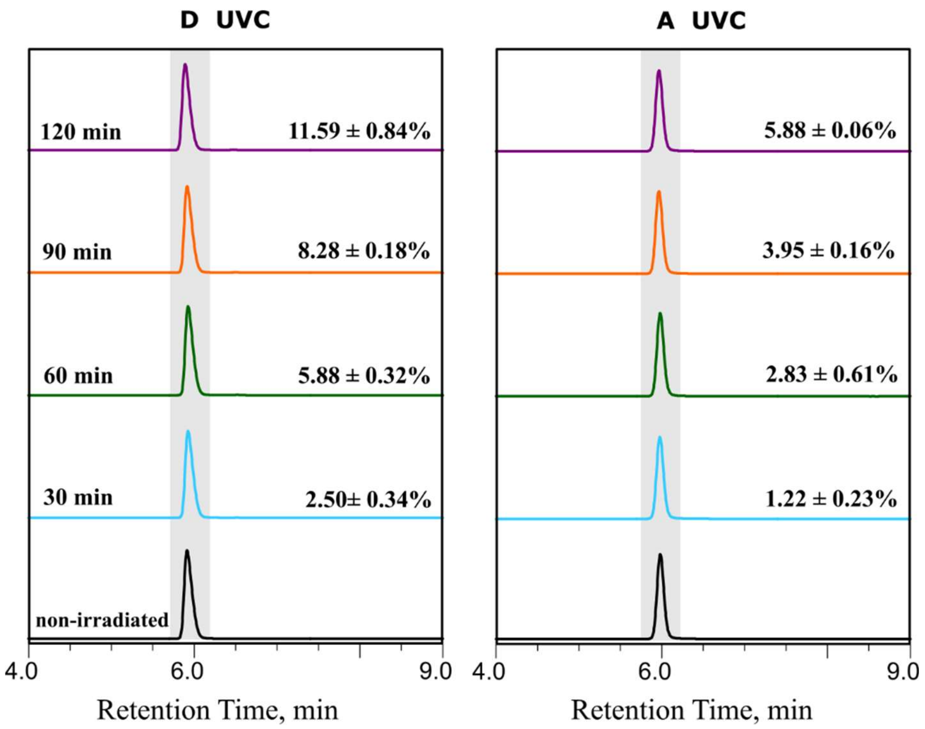

Supplementary Figure 19. Irradiation experiments in the UVC spectral range of 2,6-diaminopurine and adenine 2′-deoxyribosides (D and A). Depletion of the starting material was estimated from analytical HPLC analysis of irradiated nucleoside samples at different exposure times (between 0 and 120 minutes). Irradiations were performed at 254 nm.

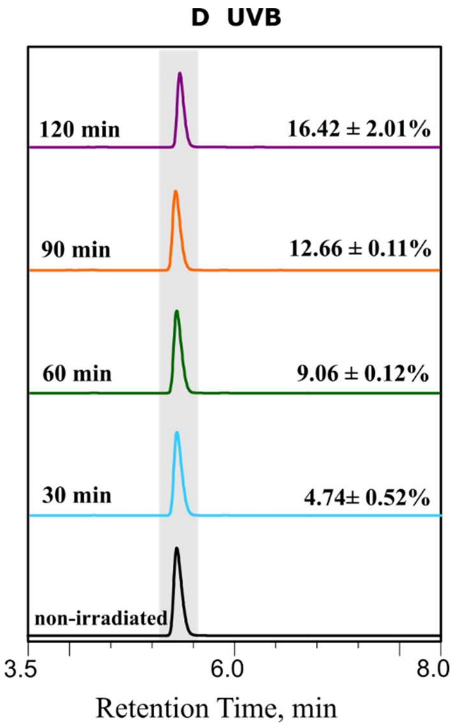

Supplementary Figure 20. Irradiation experiments in the UVB spectral range of 2,6-diaminopurine (D). Depletion of the starting material was estimated from analytical HPLC analysis of irradiated nucleoside samples at different exposure times (between 0 and 120 minutes). Adenine 2′-deoxyriboside (A) was photostable under studied irradiation conditions.

S2.2 Computational Results

Ionization energies

In order to initially estimate the electron donating properties of the D nucleoside we calculated the vertical ionization energies (VIEs) and adiabatic ionization energies (AIE) for D and the two canonical nucleosides G and A. The calculations were performed using the MP2 method and a slightly larger triple-ζ basis set (cc-pVTZ) than the one used for excited-state calculations. The VIE was obtained by calculating the energy of the radical cation on top of the minimum-energy geometry of the neutral molecule and subtracting the energy on this neutral molecule in its minimum. The AIEs were calculated simply as an energy difference between the radical cationic and neutral structures of each nucleoside in their corresponding minima. In these calculations we considered the C2'-endo conformer of the deoxyribosides with the purine base in the *syn* orientation with respect to the sugar ring and an intramolecular 5'-OH...N3 hydrogen bond. The same conformers were taken into account in the excited-state calculations (see Fig. 5 in the main article). The results contained in Supplementary Table S3 show that D has lower VIE and AIE than canonical G and A by more than 1.8 eV. This result was the first indication that D could act as an excellent electron donor and CPD repairing agent.

Supplementary Table 3. Vertical ionization energies and adiabatic ionization energies of G, A and D deoxyribosides calculated at the MP2/cc-pVTZ level of theory.

nucleoside	VIE [eV]	AIE [eV]
**G**	10.03	9.48
**A**	10.16	9.50
**D**	8.18	7.71

MD simulations and conformational analysis of damaged DNA oligomers

In order to select the most representative conformers of the studied damaged trimers we performed RMSD-based clustering of the trajectories. The trajectories simulated for AT=T in the SPC/E and OPC water model contained 3 and 4 clusters, respectively (Supplementary Figure 21). The clusters in both water models were virtually identical and only differences in populations were observed for the water models. The most common structure was the adenosine in the *syn* conformation stacked on the T=T dimer (68% and 49% in SPC/E and OPC, respectively). The *syn* conformer of the adenosine is preferred due to the presence of intramolecular 5'OH...N3 hydrogen bond. Since this interaction can only be formed at the 5' terminus, we expect that the stacked conformer with the *anti* orientation would be the dominant state in the context of oligonucleotide chains or in a double-stranded helix. The second most populated structure in both the waters had the adenine unstacked, but this cluster had a notably higher population in OPC on the expense of the stacked cluster. The other minor clusters had adenosine interacting with the edges of the T=T dimer, either with the side with two methyl groups (M), or with O2, H3 and O4 atoms at the Watson-Crick edges (W).

The trajectories of T=TA contained 7 clusters in both the SPC/E and OPC water model (Supplementary Figure 22). The identified clusters were similar in water models and mostly resembled the types of clusters found in AT=T. Due to steric restrictions given by the 5’ vs. 3’ connection in AT=T and T=TA, instead of the W-edge cluster an O-edge cluster was populated, in which the adenine was slid away from the W edge to the two sugars and O2 atoms. The relative populations of the clusters in T=TA were different from AT=T. The most common structure was an unstacked conformer with the adenosine residue not interacting with the T=T dimer and being completely exposed to the solvent. This unstacked conformer had a notably higher population in OPC. The O-edge, M-edge, and stacked clusters had approximately the same population of about 20% each. These three clusters can be found in two different forms

each, because essentially the same backbone conformation supports a major cluster with the *anti* orientation of the adenosine and a minor cluster with the *syn* orientation. This is expected as no internal 5'OH...N3 hydrogen bond could be formed in adenosine in T=TA and therefore the *anti* orientation of the adenine base was dominant over the *syn* conformation.

In addition, we performed analogous MD simulations and RMSD-based clustering for the ADT=TA pentamer. These simulations demonstrated that limited stacking of the T=T dimer with adjacent purine bases resulted in significant population of random coil structural arrangements characterized by partial stacking (~95%). Consequently, we selected one conformer, which most closely resembled structure B-DNA strand for further simulations.

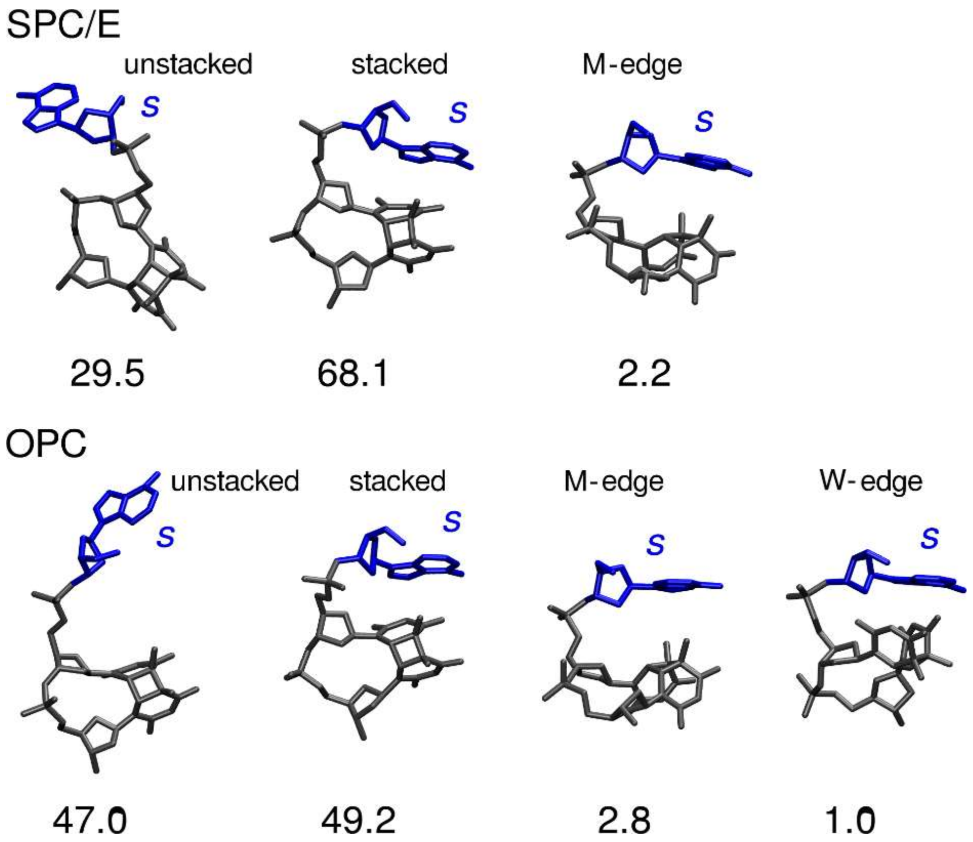

Supplementary Figure 21. Clusters centers of AT=T and the population of the corresponding cluster (%) in the SPC/E and OPC water models. Adenosine is blue and T=T dimer is grey. The blue letter "a" or "s" next to the adenosine stands for *anti* and *syn*, respectively, and denotes the conformation of its glycosidic torsion angle χ. "Unstacked", "stacked", "M-edge" and "W-edge" are cluster designations that sign the position of adenosine to the T=T dimer (see the text for details).

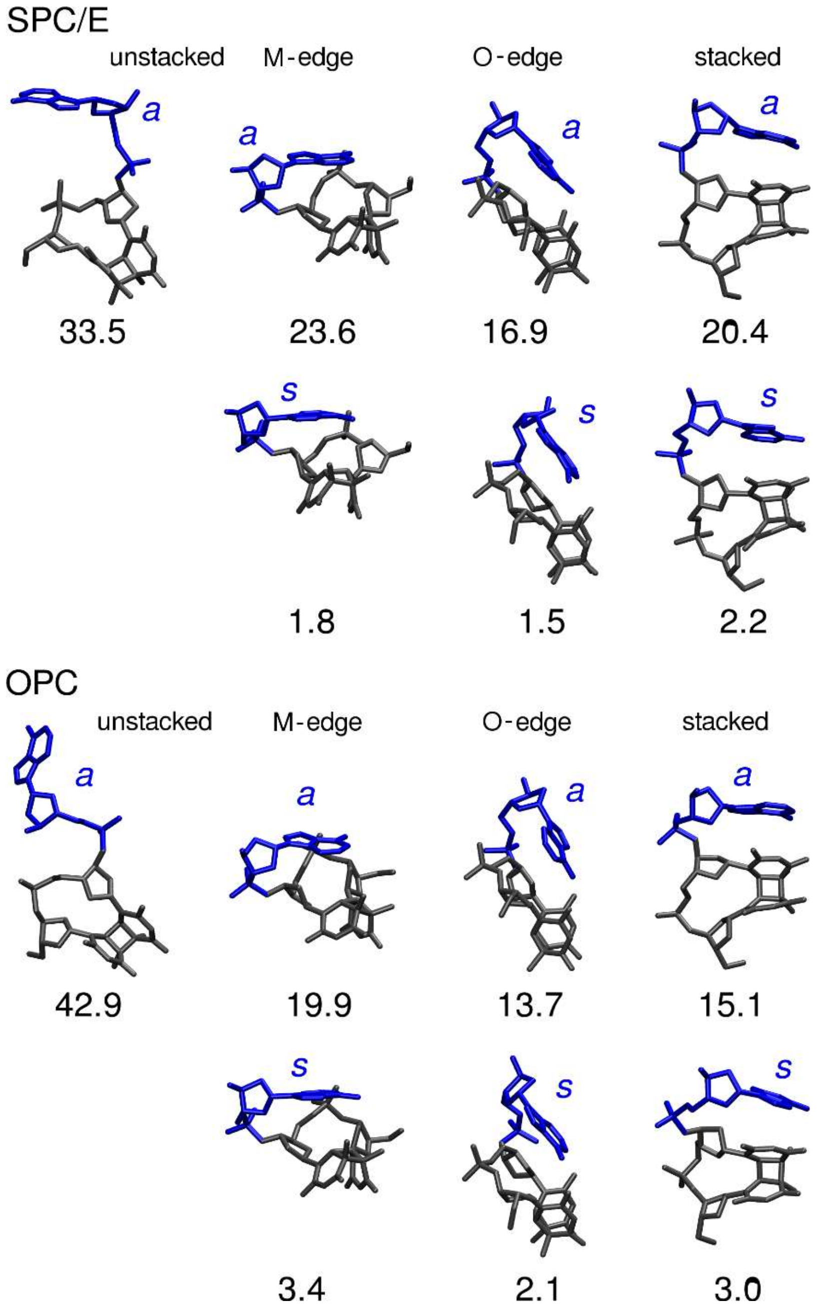

Supplementary Figure 22. Clusters centers of T=TA and the population of the corresponding cluster (%) in the SPC/E and OPC water models. Adenosine is blue and T=T dimer is grey. The blue letter "a" or "s" next to the adenosine stands for *anti* and *syn*, respectively, and denotes the conformation of its glycosidic torsion angle χ. "Unstacked", "stacked", "M-edge" and "S-edge" are cluster designations that sign the position of adenosine to the T=T dimer (see the text for details).

Ground-state geometries of the selected DNA trimers

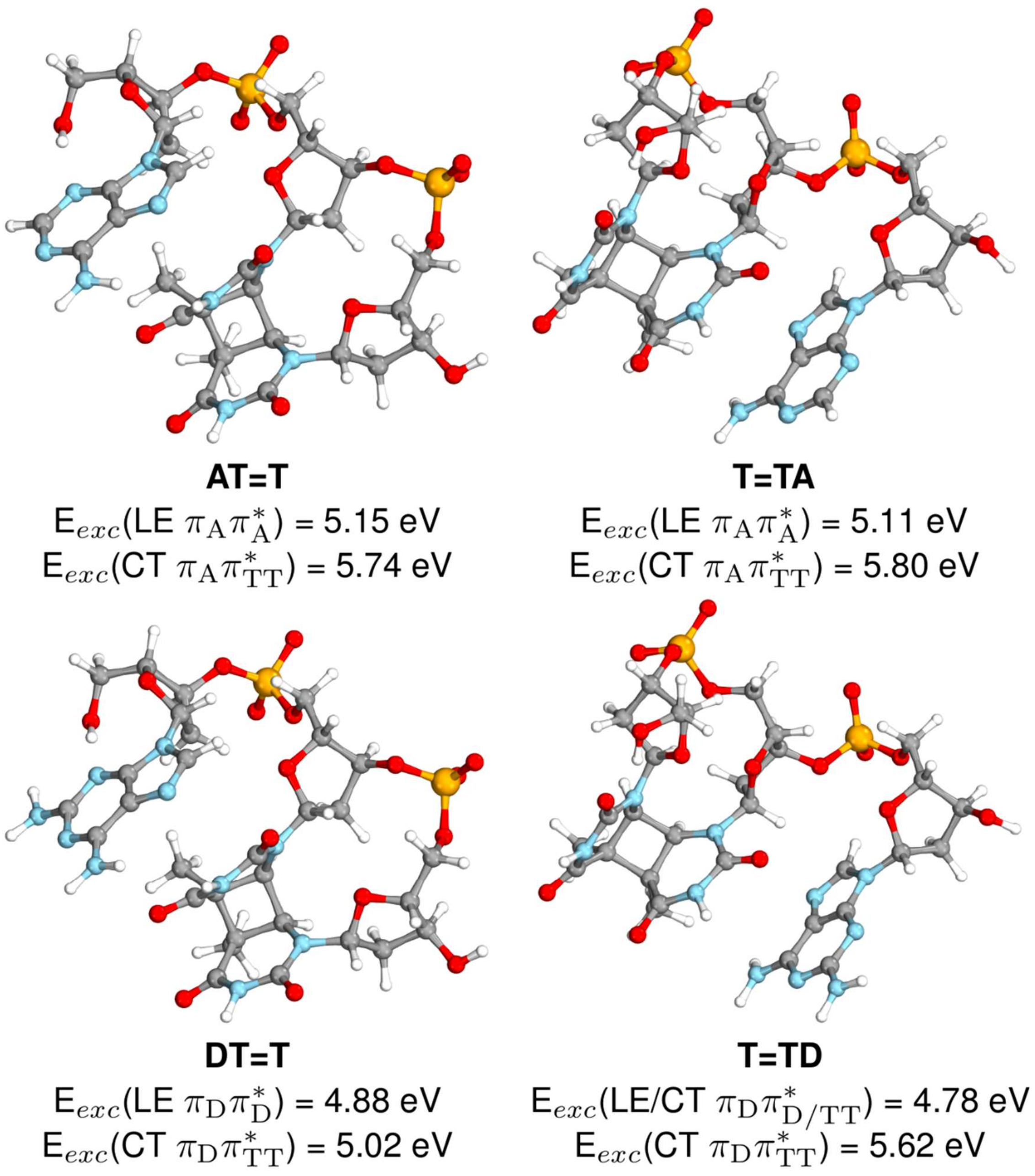

Supplementary Figure 23. Geometries of the selected trinucleotide conformers used in the excited-state calculations. The above structures were optimized using the QM_{DNA}/MM setup and the PBEh-3c. The listed key vertical excitation energies were computed at the ADC(2)/TZVP level of theory (also using the QM_{DNA}/MM setup).

Vertical excitation energies of the studied DNA trimers.

Supplementary Table 4. Vertical excitation energies of the five lowest valence states and the lowest charge transfer state of DT=T, T=TD, AT=T and T=TA calculated at the ADC(2)/TZVP level within the QM/MM framework.

State / Transition		E_{exc} [eV]	f_{osc}	λ [nm]
		DT=T		
S_1	D*T=T(ππ*)	4.88	0.169	254.3
S_2	$D^{\bullet+}T=T^{\bullet-}$	5.02	0.007	247.0
S_3	DT=T*(nπ*)	5.15	0.002	240.7
S_4	DT=T*(nπ*)	5.20	0.002	238.4
S_5	D*T=T(ππ*)	5.28	0.110	234.9
S_6	DT=T*(ππ*)	5.57	0.027	222.6
		T=TD		
S_1	T=TD*(ππ*)	4.78	0.181	259.6
S_2	T=T*D(nπ*)	5.09	0.001	243.4
S_3	T=T*D(nπ*)	5.26	0.002	235.6
S_4	T=TD*(ππ*)	5.43	0.153	228.2
S_5	$T=T^{\bullet-}D^{\bullet+}$	5.62	0.003	220.5
S_6	T=T*D(ππ*)	5.63	0.002	220.2
		AT=T		
S_1	AT=T*(nπ*)	4.93	0.001	251.4
S_2	AT=T*(nπ*)	5.08	0.001	244.0
S_3	A*T=T(ππ*)	5.15	0.215	240.5
S_4	A*T=T(ππ*)	5.27	0.096	235.1
S_5	A*T=T(ππ*)	5.40	0.019	229.5
S_7	$A^{\bullet+}T=T^{\bullet-}$	5.74	0.008	216.0
		T=TA		
S_1	T=T*A(nπ*)	4.93	0.001	251.4
S_2	T=T*A(nπ*)	5.08	0.002	244.1
S_3	T=TA*(ππ*)	5.11	0.229	242.7
S_4	T=TA*(ππ*)	5.29	0.061	234.3
S_5	T=TA*(ππ*)	5.52	0.012	224.6
S_8	$T=T^{\bullet-}A^{\bullet+}$	5.80	0.003	213.6

As observed for canonical and undamaged DNA trimers and the GAT=T tetramer, the energies of various CT states are strongly affected by the structural arrangement of the oligomer[3,4]. In particular, Lee and Matsika[4] showed that excitation energies of CT states are directly proportional to the distance between the donor acceptor pair. Similarly the difference between the vertical excitation energies of reactive CT states for the DT=T and T=TD trimers is caused by the same structural factors. In the case of the DT=T trimer having low vertical excitation energy of the CT state (5.02 eV), the Dap base is practically perfectly aligned with the C4=O carbonyl group of the neighbouring Thy base. We anticipate that the C4=O group is crucial here, since it hosts the π^* orbital responsible for accepting the transferred electron (see Supplementary Figures 23 and 24). The distance between this carbonyl oxygen and the C6 atom of Dap amounts to merely 3.18 Å for the minimum energy geometry of DT=T of the stacked conformer (Supplementary Figure 23). In contrast, the corresponding distance measured for the minimum energy structure of T=TD trimer amounts to 4.89 Å, and distances from this carbonyl oxygen to other atoms of the purine ring of Dap are even longer for T=TD. This is reflected by high vertical excitation energy of the reactive CT state in the T=TD trimer, *i.e.* 5.62 eV. However, these structural differences do not have such a strong effect on the adiabatic excitation energies of the $D^{\bullet+}T{=}T^{\bullet-}$ and $T{=}T^{\bullet-}D^{\bullet+}$ states.

Electron transfer rates calculated using a quasi-Marcus approach.

In order to qualitatively estimate the influence of substitution with D on charge transfer propensity between the purine base and T=T dimer, we calculated approximate electron transfer rates assuming the Marcus theory of electron transfer and using the following equation:

$$k = \frac{2\pi}{\hbar}|H_{AB}|^2 \frac{1}{\sqrt{4\pi\lambda k_B T}} exp\left(-\frac{(\lambda+\Delta G)^2}{4\pi\lambda k_B T}\right),$$

Where H_{ab} is the electronic coupling between donor and acceptor states, λ is the reorganization energy, ΔG is the driving force, h is the Planck constant and k_B is the Boltmzmann constant.

Here, we calculated the electronic coupling on the minimum-energy geometry of the donor state (locally excited S_1 minima of the purines) using the Boys-localization approach proposed by Subotnik *et al.*[5] The reorganization was calculated as the difference between the energy of the acceptor CT state on the minimum-energy geometry of the donor state (UV-excited purine) and the energy of the acceptor CT state in its minimum, as shown in the equation:

$$\lambda = E_{CT-state}@S_1^{purine}(min. geom.) - E_{CT-state}@S_1^{CT}(min. geom.).$$

Finally, we considered the simple energy difference between the donor and acceptor minima as the driving force in our rate constant calculations. Therefore, we denote this value in Table S5 as ΔE, since we did not perform excited-state molecular dynamics simulations to calculate the entropy contributions or generate averaged values. However, given that we used averaged structures from classical MD simulations as our starting geometries for QM/MM calculations, we expect the optimized ground and excited-state geometries to be representative. It is also worth emphasizing that all the values were calculated within the electrostatic embedding scheme, which represents the environment as point charges without any polarization effects that could contribute to the reorganization energies. Consequently, assuming all of the potential uncertainties our rate constants might not represent well the exact absolute electron transfer rates of the studied systems. However, considering the similarities between the studied trimers, we can provide a very good and qualitatively valid mechanistic rationale behind the enhanced self-repair yields of DT=T and T=TD, based on relative comparison of our estimated *k* values.

The calculated *k* values and key components used in the Marcus equation are contained in Supplementary Table 5. Considering these values, the estimated timescales of excited-state electron transfer ($t = k^{-1}$) in DT=T and T=TD amount to 141 and 260 fs respectively. This suggests that electron transfer from photoexcited D to the T=T dimer could occur on a sub-picosecond timescale and could compete with the direct radiationless photodecativation of D. In contrast, the forward electron transfer rate constant in the T=TA dimer is over an order of

magnitude lower than in T=TD, or nearly two orders of magnitude lower than in the case of DT=T. The resulting timescale of 5.82 ps is much longer that time needed for direct photorelaxation of A, which explains very low self-repair quantum yields. We were unable to calculate the electron transfer rate constant for AT=T since the CT state does not have a defined S_1 minimum, and our geometry optimizations of this state resulted in the stabilization of the locally-excited ππ* configuration on the T=T dimer. However, considering that the driving force (represented as ΔE in Table S5) is the key element that influenced the difference in *k* values for the three trimers, we expect the electron transfer rate of UV-excited AT=T to be even lower.

Supplementary Table 5. Key components used for the calculation of electron transfer rate constants within the quasi-Marcus approach outlined above.

Trinucleotide	H_{ab} [eV]	ΔE [eV]	λ [eV]	k [s^{-1}]
DT=T	$3.93\cdot10^{-2}$	-1.47	3.06	$7.08\cdot10^{12}$
T=TD	$5.85\cdot10^{-2}$	-1.53	3.68	$3.85\cdot10^{12}$
T=TA	$9.34\cdot10^{-2}$	-0.11	2.82	$1.72\cdot10^{11}$

Characteristics of the CT states in DNA trimers

Molecular orbitals associated with the key reactive CT states are presented in Supplementary Figure 24. These configurations have the dominant contribution (over 85%) to the molecular orbital character of these states. Therefore they also accurately illustrate the charge redistribution in the excited states responsible for self-repair of these trimers. We additionally performed particle-hole analysis using the TheoDore 1.5.1 program to estimate the amount of charge transferred between the purine and T=T fragments. These calculations indicate that for each of the three trimers (DT=T, T=TD and T=TA) the amount of charge transferred between these two fragments is between 0.99 and 1.0 electron. We did not perform the corresponding analysis for the AT=T owing to lack of a defined CT minimum in the S_1 state (the CT minimum is higher in energy).

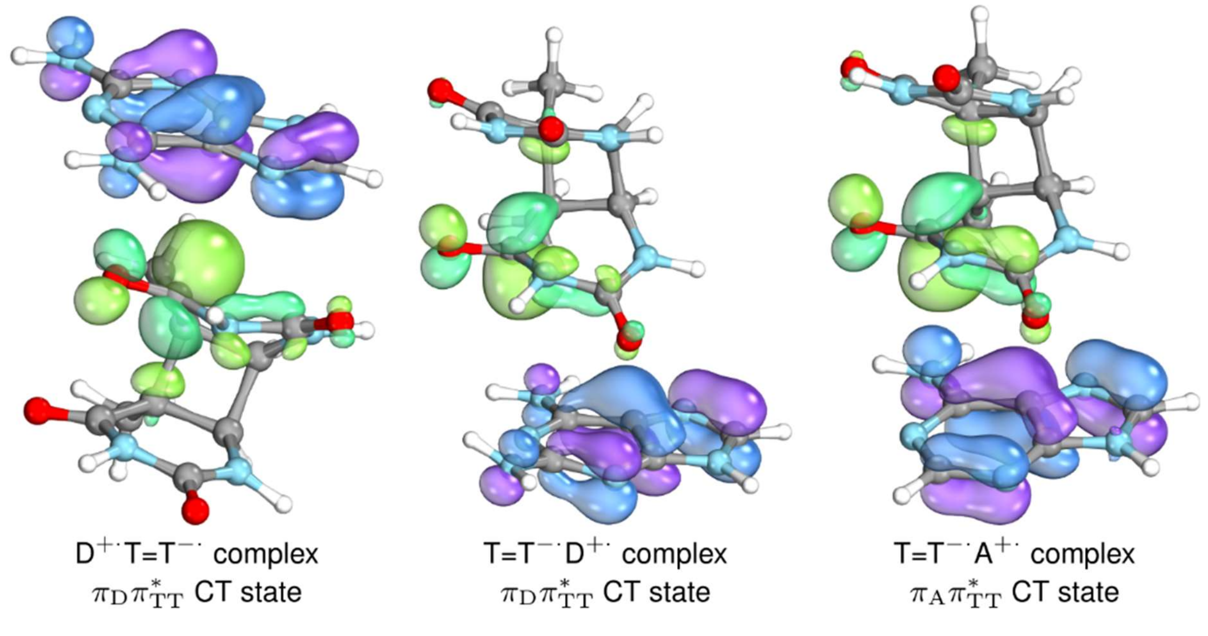

Supplementary Figure 24. Minimum energy geometries of the S_1(CT) states of the damaged DT=T, T=TD and T=TA trinucleotides. The blue and purple molecular orbitals correspond to the occupied π orbitals on the purine bases which acts as the electron donor, while the light/dark green molecular orbitals correspond to the virtual π* orbitals on the T=T dimers, acting as the electron acceptor site.

Opening of the T=T dimer after photoinduced electron transfer

To estimate the energy barrier associated with the breaking of the C5-C5 bond in the reactive CT state we calculated a relaxed PE scan along this particular bond distance using the QM_{bases}/MM setup, the ADC(2) QM method and the smaller SV(P) basis set (see Supplementary Figure 25). In these calculations the C5...C5 distance was kept frozen, while all the remaining degrees of freedom were optimized. The calculations performer for the DT=T damaged trimer are presented in Supplementary Figure 25. Similarly as in the case of our previous study focused on the self-repair of the GAT=T tetramer, the opening of the cyclobutane ring in the reactive $D^{\bullet +}T{=}T^{\bullet -}$ state is associated with a very modest barrier, which amounts to merely 0.15 eV (3.5 kcal/mol; 14.5 kJ/mol), a value that it typical for conformational changes. Once the C5...C5 distance is extended beyond 1.82 Å, further elongation is driven by the steepness of the S1 PE surface towards a peaked S_1/S_0 conical intersection. This state crossing is reached when the C5...C5 distance reaches 2.42 Å. At this point, the system can effectively interconvert to the electronic ground state in which, the C6-C6 may be broken in a barrierless way. Analogous calculations performed for the T=TA triner in the reactive $T{=}T^{\bullet -}A^{\bullet +}$ state returned an identical energy barrier of 0.15 eV. Therefore, once the electron is transferred from the UV-excited purine base to the T=T CPD, the photochemical ring opening as practically spontaneous process.

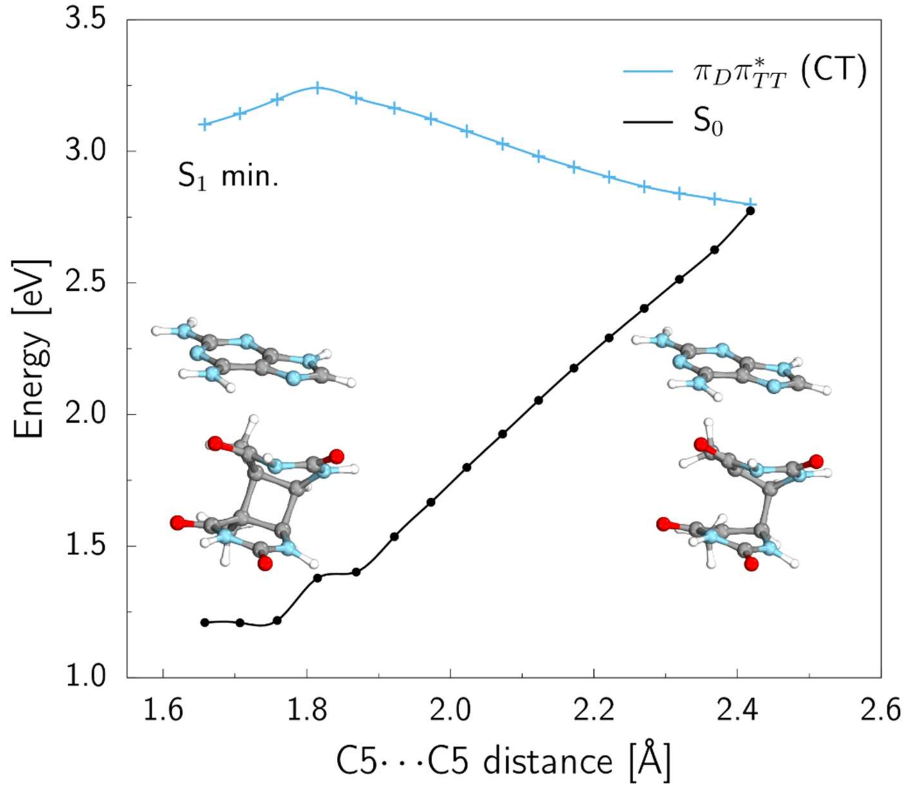

Supplementary Figure 25. Relaxed potential energy profile calculated for the C5...C5 distance in the reactive $D^{\bullet+}T{=}T^{\bullet-}$ state. The QM_{bases}/MM setup was used with the ADC(2)/SV(P) QM approach.

Excited-state QM/MM calculations for the ADT=TA pentamer

In addition to the excited-state QM/MM calculations of damaged DNA trimers, we considered a simple model system, which mimics an internally positioned CPD lesion near the Dap base. We first optimized the ground-state minimum energy structure of a well-stacked ADT=TA conformer selected based on prior MD simulations using the PBEh-3c composite hybrid DFT method[20] within the QM_{DNA}/MM setup (see Supplementary Figure 26 and Figure 7 in the main article). We then calculated the vertical excitation energies applying the QM_{bases}/MM setup and the ADC(2)/def2-SVP method. The energy of the lowest excited singlet state for this geometry amounts to 4.90 eV and the character of this state is dominated by the CT configuration with an electron transferred from D to the T=T dimer ($\pi_D\pi_{TT}^*$) mixed with local $\pi_D\pi_D^*$ excitation on the D chromophore (see orbitals in Supplementary Figure 26). In order to validate this result on the full DNA fragment, we calculated the vertical excitation energies using the QM_{DNA}/MM scheme and the ADC(2) with a the somewhat smaller SV(P) basis set because of increased computational costs for the larger QM region. This calculation yielded the excitation energy of the S_1 state equal to 4.88 eV with the same electronic configurations having a major contribution to the character. We further optimized the $AD^{\bullet+}T{=}T^{\bullet-}A$ and AD*T=TA S_1 minima using the QM_{bases}/MM partitioning scheme and the ADC(2)/def2-SVP method. These geometry optimizations indicate that the $AD^{\bullet+}T{=}T^{\bullet-}A$ minimum lies 0.72 eV lower in energy than the AD*T=TA minimum. Consequently, charge transfer from D to T=T is an energetically favorable photorelaxation pathway and it may occur immediately upon the photoexcitation of the ADT=TA pentamer. Similar as in the case of the studied DNA trimers, electron transfer will subsequently enable breaking of the C5-C5 bond and CPD self-repair.

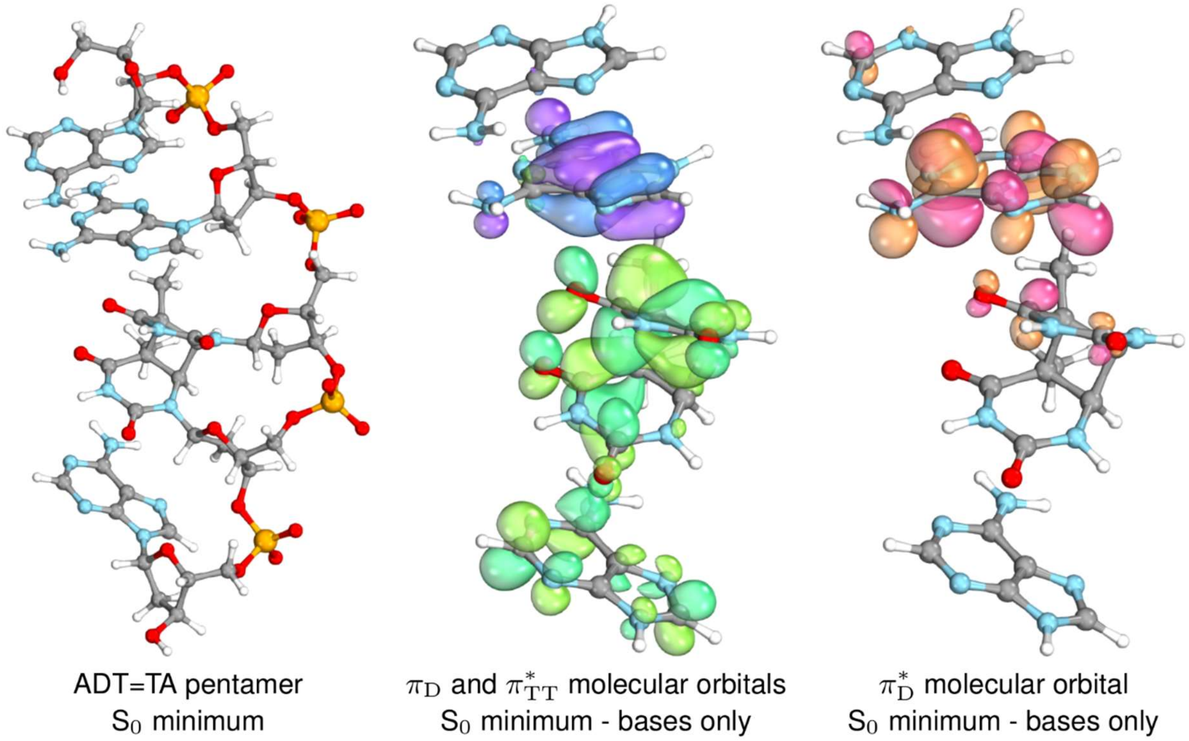

Supplementary Figure 26. Minimum-energy S_0 geometries (optimized using the PBEh-3c method and QM_{DNA}/MM scheme) of the selected stacked conformer of the ADT=TA pentamer. Middle and right figures show the nucleobases only and the key molecular orbitals, which characterize the lowest excited singlet state: blue and purple - π_D; green - π_{TT}*; red - π_D*.

Supplementary References

1. Pan, Z., Chen, J., Schreier, W. J., Kohler, B. & Lewis, F. D. Thymine Dimer Photoreversal in Purine-Containing Trinucleotides. *J. Phys. Chem. B* **116**, 698–704 (2012).
2. Roberts, S. J. et al. Selective prebiotic conversion of pyrimidine and purine anhydronucleosides into Watson-Crick base-pairing arabino -furanosyl nucleosides in water. *Nat. Commun*. 9, 4073 (2018).
3. Szabla, R., Kruse, H., Stadlbauer, P., Sponer, J. & Sobolewski, A. L. Sequential Electron Transfer Governs the UV-Induced Self-Repair of DNA Photolesions. *Chem. Sci.* **9**, 3131-3140 (2018).
4. Lee, W. & Matsika, S. Role of charge transfer states into the formation of cyclobutane pyrimidine dimers in DNA. *Faraday Discuss.* **216**, 507–519 (2019).
5. Subotnik, J. E., Yeganeh, S., Cave, R. J. & Ratner, M. A. Constructing diabatic states from adiabatic states: Extending generalized Mulliken–Hush to multiple charge centers with Boys localization. *J. Chem. Phys.* **129**, 244101 (2008).
